# Cloning of *Gossypium hirsutum* Sucrose Non-Fermenting 1-Related Protein Kinase 2 Gene (*GhSnRK2*) and Its Overexpression in Transgenic *Arabidopsis* Escalates Drought and Low Temperature Tolerance

**DOI:** 10.1371/journal.pone.0112269

**Published:** 2014-11-13

**Authors:** Babatunde Bello, Xueyan Zhang, Chuanliang Liu, Zhaoen Yang, Zuoren Yang, Qianhua Wang, Ge Zhao, Fuguang Li

**Affiliations:** State Key Laboratory of Cotton Biology, Cotton Research Institute, Chinese Academy of Agricultural Sciences, Beijing, China; University of Delhi South Campus, India

## Abstract

The molecular mechanisms of stress tolerance and the use of modern genetics approaches for the improvement of drought stress tolerance have been major focuses of plant molecular biologists. In the present study, we cloned the *Gossypium hirsutum* sucrose non-fermenting 1-related protein kinase 2 (*GhSnRK2*) gene and investigated its functions in transgenic Arabidopsis. We further elucidated the function of this gene in transgenic cotton using virus-induced gene silencing (VIGS) techniques. We hypothesized that *GhSnRK2* participates in the stress signaling pathway and elucidated its role in enhancing stress tolerance in plants via various stress-related pathways and stress-responsive genes. We determined that the subcellular localization of the *GhSnRK2*-green fluorescent protein (GFP) was localized in the nuclei and cytoplasm. In contrast to wild-type plants, transgenic plants overexpressing *GhSnRK2* exhibited increased tolerance to drought, cold, abscisic acid and salt stresses, suggesting that *GhSnRK2* acts as a positive regulator in response to cold and drought stresses. Plants overexpressing *GhSnRK2* displayed evidence of reduced water loss, turgor regulation, elevated relative water content, biomass, and proline accumulation. qRT-PCR analysis of *GhSnRK2* expression suggested that this gene may function in diverse tissues. Under normal and stress conditions, the expression levels of stress-inducible genes, such as *AtRD29A, AtRD29B, AtP5CS1, AtABI3, AtCBF1*, and *AtABI5*, were increased in the *GhSnRK2*-overexpressing plants compared to the wild-type plants. *GhSnRK2* gene silencing alleviated drought tolerance in cotton plants, indicating that VIGS technique can certainly be used as an effective means to examine gene function by knocking down the expression of distinctly expressed genes. The results of this study suggested that the *GhSnRK2* gene, when incorporated into Arabidopsis, functions in positive responses to drought stress and in low temperature tolerance.

## Introduction

Plants have developed complex signaling pathways in response to various environmental stresses, such as salt, drought, and cold, and have acquired metabolic functions and developmental methods to survive changing environmental conditions [1]. Improving crop resistance to drought stress would be the most valuable means to improve agricultural productivity and to reduce crop loss caused by environmental stress. As a result, understanding the mechanisms of drought tolerance and developing drought-resistant crop plants have been major targets of plant molecular biologists and biotechnologists. Low-temperature constraints have been progressively overcome by the recognition of cold-tolerant genes for applications in transgenic plants. Transgenic approach has reveal many possibilities to improve cold stress in plants by incorporating or deleting genes that regulate a particular characteristic [2]. These approaches also provide unique opportunities to improve the genetic quality of plants via the development of particular crop varieties that exhibit enhanced resistance to biotic and cold stresses and improved nutritional quality. The plant response to salt stress typically results in osmotic alterations, which play a major role in ensuring osmotic balance in plant cells. During plant stress responses, the regulation of gene expression involves both universal and unique changes in the transcript levels of certain plant genes [3]. Plants directly or indirectly respond to stresses by initiating signal transduction pathways. Various abiotic stresses result in both general and specific effects on plant growth and development. For example, drought limits plant growth due to difficulties in maintaining turgor pressure, photosynthetic decline, osmotic stress-imposed constraints on plant processes and interference with nutrient availability as the soil dries [4].

Protein kinases and phosphatases are major elements of stress signals which are transmitted to different cellular regions via specialized signaling pathways. Some of the protein kinases involved in stress signal transduction in plants, such as mitogen-activated protein kinases (*MAPKs*) [5], [6], [7], [8], glycogen synthase kinase 3 (*GSK3*) [9], [10], and S6 kinase (*S6K*) [11], are similar among all eukaryotic organisms, whereas others, including calcium-dependent protein kinases (*CDPKs*) [12], [13], [14] and *SNF1*-related kinases (*SnRKs*), are plant-specific.

*SnRK2* is an important stress-related protein kinase in plants that has been implicated in stress and abscisic acid-mediated signaling pathways [15]. Previously, it was reported that *SRK2C/SnRK2.8*, a subclass II member, was strongly activated by drought stress and that plants overexpressing *SRK2C* exhibited improved drought tolerance as a result of the up-regulation of many stress-inducible genes [16]. *SnRK2s* have a molecular weight of approximately 40 kDa and are monomeric serine/threonine protein kinases [17], [18]. Based on phylogenetic analysis, three groups of *SnRK2* family members have been identified. Group 1 consists of kinases not activated by ABA, group 2 consists of kinases not activated or activated very weakly by ABA, and group 3 consists of kinases strongly activated by ABA. The amino acid sequences of all *SnRK2s* can be separated into two regions, the highly conserved N-terminal kinase domain and the regulatory C-terminal domain, which contains stretches of acidic amino acids. Furthermore, the C-terminal domain consists of two subdomains, Domain I and Domain II. Domain I is characteristic of all *SnRK2* family members and is required for activation by osmotic stress. Based on our investigation, we found that the expression of *GhSnRK2* was induced by PEG. PEG was quite commonly used in physiological experiments to induce controlled drought stress. In the present study, we generated a gene construct containing *GhSnRK2* driven by the constitutive cauliflower mosaic virus (CaMV) 35S promoter and transformed this construct into *Arabidopsis* in order to investigate the functional analysis of *GhSnRK2* gene. We monitored the activities of this gene with respect to drought and low temperature tolerance in transgenic plants. We further elucidated the function of this gene in transgenic cotton using virus-induced gene silencing (VIGS) techniques. We showed that *GhSnRK2*, the cotton *SnRK2* gene, is involved in multi-stress responses. Our results promote the analysis of gene function in *G. hirsutum* to facilitate the exploitation of desirable genes from this species. The elucidation of *GhSnRK2* gene function contributes to our understanding of the mechanism by which this plant adapts to abiotic stress and provides a valuable gene resource for plant breeders.

## Materials and Methods

### Cloning of theGhSnRK2 gene

Total RNA was extracted from CCRI24 cotton tissues using Trizol (Sigma-Aldrich) according to the manufacturer's instructions. Reverse transcription (RT) was performed using total RNA extracted from seedlings oligo(dT)16 primer, and SuperScript II reverse transcriptase (Promega). The RT product was used in PCRs to amplify the predicted *GhSnRK2* open reading frame using primer star polymerase (enzymes). The cDNA regions of *GhSnRK2* were cloned into the T-simple vector. All of the clones were confirmed via sequencing. Primers specific to the sequence of *GhSnRK2* were designed, synthesized, and used to clone the *SnRK2* gene. For plant transformation, *GhSnRK2* cDNAs were introduced into the modified pCAMBIA2301 plant transformation vector under the control of the CaMV 35S promoter.

### Localization of the *GhSnRK2*-GFP fusion protein

The method of [19] was adopted to perform the subcellular localization assay. The *GhSnRK2* coding sequence was cloned and ligated into the XbaI and SpeI sites of the PCAMBIA2301-GFP vector to generate PCAMBIA2301-*GhSnRK2*-GFP, which expressed the *GhSnRK2*-GFP fusion protein under the control of the CaMV35S promoter. The construct was used for transient transformation of onion. Onion epidermal peels were bombarded with DNA-coated gold particles, and GFP expression was visualized 24 h later. Transformed onion cells were observed under a confocal microscope.

### Effect of polyethylene glycol (PEG) treatment on *GhSnRK2* gene expression

The effect of PEG on the expression level of the *GhSnRK2* gene was evaluated via qRT- PCR. The root of a three-week-old upland cotton plant was submerged in 10% PEG solution, and samples were collected from the root at one hour intervals for 6 hours. RNA was extracted from the samples, and RT was performed as described above to generate cDNA for qRT- PCR analysis.

### *Arabidopsis* transformation and screening of transgenic plants

pCAMBIA2301 carrying *GhSnRK2* was introduced into *Agrobacterium tumefaciens* strain GV3101. The transgenic *Arabidopsis* plants were generated using the flower dipping method [20], and transgenic plants were selected based on their growth in 0.8% agar containing half-strength MS salts and kanamycin. The transformants were transferred to soil and allowed to set seed. The T3 generation of the transgenic plants was used for all experiments.

### Plant materials and growth conditions

*Arabidopsis thaliana* seeds and ecotype Col-0 wild-type, mutant, and transgenic seeds were surface-sterilized using bleach (5% Sodium hypochlorite) and 0.1% Triton X-100. After cold exposure at 4°C for 2 d, the seeds were germinated and cultured in plates containing 0.5× MS medium, 0.8% agar, and 1% sucrose under continuous light at 22°C. For plants grown in soil, 7-day-old seedlings were transferred from the MS plates to soil and cultured under 80–100 µmol m^−2^s^−1^ photoperiodic cycles of 16 h light and 8 h dark at 22°C in a growth chamber under fluorescent light using one cool-light and one warm-light tube, each of which was suspended several inches above the plants. Mature seeds obtained from transformed plants (T_0_ generation) were cultured in MS medium containing the antibiotic kanamycin in order to screen for transformed plants. The seeds were allowed to grow in the medium for two weeks before transfer to soil for growth continuation. Cotton seeds were planted in moist soil and grown in a growth chamber with a 14 h photoperiod at a 20/30°C night/day temperature cycle, with a light intensity of 400 µmolm^−2^s^−2^ and at 60% relative humidity.

### Drought tolerance assay

Drought stress tolerance was measured by transferring 2-week-old plants cultured in Petri dishes to pots (10-cm diameter) filled with a 1∶1 (v/v) vermiculite:perlite mixture. The seedlings were cultured for 2 weeks with constant watering before the water was withheld. After 9 days without water, all of the pots were re-watered simultaneously, and plant re-growth was scored 4 days later. Thirty-five plants for each individual transgenic line were used in each repeated experiment, and one representative image is shown. Plants were scored as survivors if there were healthy green young leaves after re-watering treatment. The survival rate was calculated as the ratio of number of surviving plants to the total number of treated plants in the flower pot. Representative transgenic and WT plants of were photographed before and after drought treatment. Each stress assay was repeated at least three times. The water use efficiency (WUE) of the plants was calculated as the amount of water needed to maintain the weight of each experimental pot containing the plants. To determine the soil water content (SWC), the pots were immediately weighed after saturation with water prior to the initiation of drought stress and then periodically weighed during the drought stress period. The SWC was calculated as (final fresh weight-dry weight)/(initial weight–dry weight)x100. The plants were exposed to water stress by reducing the water content to 25 to 30% of the SWC. For plant tugor pressure assay, drought stressed plants were removed from the growth chamber and transferred to a dark laboratory cupboard for 4 h before turgor measurements using the pressure chamber [21]. To measure the drought tolerance of *GhSnRK2* gene silenced and non-silenced plants, water was withheld for 5 days, approximately two weeks post-inoculation, and the survival rate was recorded as the percentage of plants that survived after re-watering for 3 days. Each of the ten treated groups consisted of five plants.

### Freezing and cold stress treatment

Transgenic plants overexpressing the *GhSnRK2* gene and wild-type *Arabidopsis* plants were cultured in pots filled with a 1∶1 mixture of perlite and vermiculite under a long-day photoperiod at 22°C. Freezing stress was performed by transferring 4-week-old plants to a chamber in which the temperature was adjusted to −4°C and −8°C for 10 h, respectively, and then returned to the typical standard growth conditions. For cold stress, 4-week-old plants were transferred to 4°C for three weeks. The plants were analyzed after recovery for 7 days under normal growth conditions. The survival rate (%) under freezing and cold conditions was calculated as the percentage of clearly green plants after returning to normal conditions, and plants exhibiting clear sign of wilting were denoted as dead. Thirty-five plants for each individual line were used in each repeated experiment, and one representative image is shown.

### Transpirational water loss assay

The leaves of mutant and wild-type seedlings at the rosette stage were detached and placed in a weighing boat, and the changes in fresh weight over time were monitored using an electronic balance. The rate of water loss was calculated as the loss in fresh weight of the samples. Six plants of each transgenic and WT line were analyzed in this assay, and this experiment was replicated three times [22].

### Relative water content

The relative water content of the leaves was measured according to the method of [23]. Fully expanded leaves were cut from the plants, and the fresh weight (FW) was recorded immediately. Then, the fresh portions were immersed in distilled water for 4 h and the turgid weight (TW) was recorded. Finally, the dry weight (DW) was recorded after drying for 48 h at 80°C in an oven. The RWC was calculated according to the following formula: RWC (%) = (FW−DW)/(TW−DW)×100.

### Measurement of stomatal closure in response to ABA treatment

Stomatal closure assays were conducted as described by [24]. Leaves from *GhSnRK2* transgenic and WT plants were immersed in a solution containing 50 µM CaCl_2_, 10 mM KCl, and 10 mM 2-(N-morpholino) ethanesulfonic acid (MES)-Tris, pH 6.15, and were exposed to light for 2 h. Subsequently, various concentrations of ABA were added to the solution. The stomatal apertures were measured after 2 h of ABA treatment.

### Determination of proline content

The free proline content was measured using the method described by [25]. Leaf segments were homogenized in 3% sulfosalicylic acid, and the homogenates were centrifuged at 3000 g for 20 min. Mixtures containing 2 ml of sample supernatant, 2 ml of acetic acid, and 2 ml of 2.5% acid ninhydrin solution were boiled for 30 min, and the absorbance at 520 nm (A520) was measured.

### Chlorophyll content assay

The determination of chlorophyll content was performed according to the method of [26]. Extracts were obtained from 0.1 g (fresh weight) leaf samples from 4-week-old plants and were homogenized in 1 ml of 80% acetone to quantify the chlorophyll content via spectrophotometric analysis.

### Seedling growth in response to ABA and NaCl treatment

The sensitivity of seedling germination to ABA and NaCl was assessed on MS agar plates containing various concentrations of ABA and NaCl solution [27]. Seedlings from WT and transgenic plants were placed on MS agar plates supplemented with distilled water or different concentrations of ABA or NaCl and were placed in the growth chamber at 22°C.

### Germination assay

To evaluate seed germination, the method of [27] was employed. Seeds from wild-type and transgenic plants were surface-sterilized using sodium hypochlorite and were placed on Murashige and Skoog (MS) solid medium containing various concentrations of ABA (0 µm, 0.3 µm, or 0.5 µm). For the evaluation of seed germination under salt stress, the MS medium was supplemented with various concentrations of NaCl (0 mM, 50 mM, or 100 mM). The percentage of germinating seeds was recorded after 7 days.

### Biomass accumulation

Plants were harvested for biomass measurements after 4 weeks of germination. The fresh weight of each individual plant shoot and root was measured immediately after harvesting. For fresh weight biomass, the fresh weight of the root and the shoot was measured immediately after harvesting, and the dry weight was recorded in (g) after drying in an oven to a constant weight at 70° for 48 h.

### qRT-PCR

Total RNA was extracted from *Arabidopsis* seedlings using Trizol reagent (Takara) and was treated with RNase-free DNase (Promega). cDNA was synthesized using a Promega kit according to the manufacturer's protocol. qPCR was performed using a SYBR green PCR master mix kit. After PCR, the data was quantified using the comparative CT method (2^−^ΔΔCT method) based on the CT values [28]. *AtACT2* (gene accession At3G18780) and cotton *histone*3 (gene accession AF024716) were used as internal controls, and the relative expression level of each target gene was quantified. Each RT-PCR measurement was performed in triplicate. All RT-PCR experiments were reproduced at least three times using independent cDNA samples.

### Silencing construct development and cotton transformation

The method described by [29] was employed to construct the VIGS vector. Specific fragments (approximately 300 bp) were amplified via PCR and cloned into the T-simple vector according to the manufacturer's specifications (Promega). The resulting clones were sequenced. The plasmids were digested using the restriction enzymes Xba1 and BamH1 and were subsequently ligated into the VIGS vector (*PYL156:(pTRV-RNA2)-GhSnRK2*). The resulting constructs were named according to the putative function of the stress-related gene. For transformation, *Agrobacterium* (GV3101) carrying *GhSnRK2* derivatives were cultured at 28°C in LB medium containing appropriate antibiotics. Cells were harvested from cultures grown overnight, re-suspended in inoculation buffer (200 µM acetosyringone, 10 mM MES, pH 5.5), and incubated for 2 h at room temperature in a shaker. *Agrobacterium* strains containing the *GhSnRK2* derivative *PYL156:(pTRV-RNA2)-GhSnRk2*, *PYL192:(pTRV-RNA1)*, *PYL156:(pTRV-RNA2)-GrCLA1*(positive control), or*PYL156:(pTRV-RNA2)* vector (OD 1.5) were mixed at a 1 : 1 ratio in 5 mM MES buffer (pH 5.5) and inoculated into *Gossypium hirsutum* leaves using a needleless syringe. The inoculated plants were maintained in a green house at 23±2°C for effective viral infection and spreading.

### Experimental design and statistical analysis

The data obtained were subjected to statistical analysis and were expressed as the means and standard error (SE) of at least three replicates. All experimental data are presented as the means of at least three independent replicates, and the analysis for significance was performed using Student's T-test.

The sequences of primers used in this study are listed in Table S2.

## Results

### Phylogenetic analysis, subcellular localization, and expression pattern of *GhSnRK2* in cotton plant

**Figure 1:**
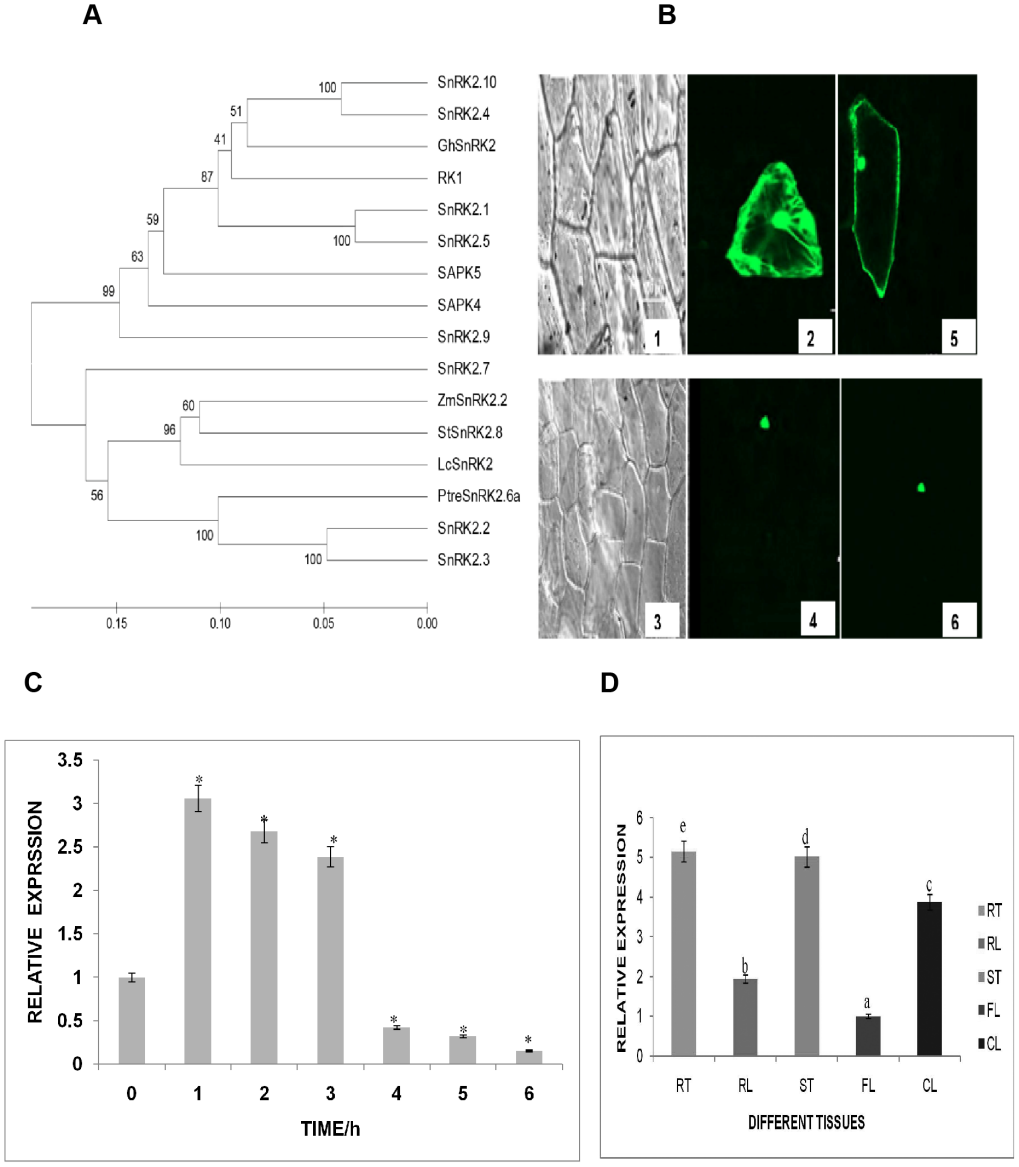
Phylogenetic analysis, subcellular localization, and expression pattern of *GhSnRK2* in cotton plant. (A) A phylogenetic tree of *GhSnRK2* and other *SnRK2* proteins from different plants was constructed using the neighbor-joining method with MEGA 5. The sequences used for analysis are listed by accession number: *Litchi chinensis* (*LcSnRK2*), AFX72761.1; *Arabidopsis thaliana* (*AtSNRK2.2*), CP002686.1; *Arabidopsis thaliana* (*AtSnRK2.3*), AED98274.1; *AtSnRK2.1*, AED91326.1; *AtSnRK2.9*, AEC07398.1; *AtSnRK2.10*, AEE33751.1; *AtSnRK2.4*, AEE28666.1; *AtSnRK2.5*, AED97781.1; *AtSnRK2.7*, AEE87152.1; *Populus tremula* (*PtreSnRK2.6a*), AGW51610.1; *Zea mays* (*ZmSnRK2.2*), NM_001137717.1; *Solanum tuberosum* (*StSnRK2.8*), AFR68945.1; *Oryza sativa* (*RK1*), ABB89146.1; *Oryza sativa* (*SAPK4)*, BAD18000.1; *Sorghum bicolor* (*SAPK4*), AGM39623.1; and *Zea mays* (*SAPK5*), ACG42286.1. The bootstrap values are shown on the tree branches. (B) Subcellular localization of the *GhSnRK2-GFP* protein. (2 and 5) GFP alone; (4 and 6) *GhSnRK2-GFP* in onion epidermal cells; (1 and 3) corresponding bright-field images. (C) The expression pattern of the *GhSnRK2* gene in cotton plants subjected to 10% PEG stress. The gene expression data were normalized to that of the cotton histone 3 gene. The values are presented as the means of three experimental replicates. The vertical axis represents the relative expression level. The values from1 to 6 indicate the time (h) of PEG treatment. Asterisk denotes a significant difference (P<0.05) compared with the control (0 h). (D) Relative expression levels of the *GhSnRK2* gene in various cotton plant tissues. Samples from root (RT) stem (ST), cauline leaves (CL), rosettes leaves (RL) and flowers (FL) were analyzed. The vertical axis represents the relative expression level. The letters denote significant differences (P<0.05) based on Duncan's multiple range tests. The cotton histone 3 gene was used as an internal control for normalization of the gene expression data.

The phylogenetic tree constructed using the full length amino acid sequence of selected *SnRK2* genes to analyze the evolutionary relationship between *GhSnRK2* and other *SnRK2* family genes is shown in the neighbor-joining tree developed based on an alignment of the complete protein sequences. The bootstrap values are shown on the branches. *GhSnRK2* clustered with the known stress-related genes *AtSnRK2.1*, AED91326.1; *AtSnRK2.4*, AEE28666.1; *AtSnRK*2.5, AED97781.1; *AtSnRK2.10*, AEE33751.1; and *Oryza sativa* (*RK1*), ABB89146.1. *SnRK2.10* and *SnRK2.4* are closely related to *GhSnRK2*, suggesting that *GhSnRK2* belongs to the *SnRK2* family (Figure 1A). Alignment of the *GhSnRK2* amino acid sequence with that of other *SnRK2s* revealed that the *GhSnRK2* protein is highly similar to other *SnRK2s*; the relatively conserved motif is underlined. The deduced amino acid sequence displays relatively high homology with the monocot *SnRK2* family members *Oryza sativa* (*RK1*), ABB89146 and with the dicot species *AtSnRK2.10*, AEE33751.1 (Figure S1A). The *GhSnRK2*-GFP fusion protein driven by the CaMV 35S promoter was transiently expressed in onion epidermal cells, and the green fluorescent *GhSnRK2* protein signals were localized to the nuclei and the cytoplasm, whereas GFP alone was detected throughout the cell (Figure1B). qRT-PCR was performed to quantify the expression level of the *GhSnRK2* gene. Treatment of 3-week-old CCRI24 upland cotton cultivar plants with 10% PEG for different periods induced the expression of the *GhSnRK2* gene for 3 h, after which the expression level declined (n = 3) (Figure 1C). To investigate the distribution of *GhSnRK2* in different tissues, samples from root (RT), stem (ST), cauline leaves (CL), rosette leaves (RL) and flowers (FL) were analyzed. *GhSnRK2* gene expression was detected in all of the examined tissues in distinct expression patterns and was highest in the root and lowest in the flower, suggesting that the *GhSnRK2* gene may most actively function in the root (Figure 1D). Cotton *histone*3 (gene accession AF024716) was used as internal control to normalize expression data. The values are presented as the means of three replicates, and the error bars denote the SE. Different letters denote the means ± standard deviation displaying significant difference at P≤0.05.

### Plant transformation vector and the expression pattern of the *GhSnRK2* gene in the transgenic lines

**Figure 2:**
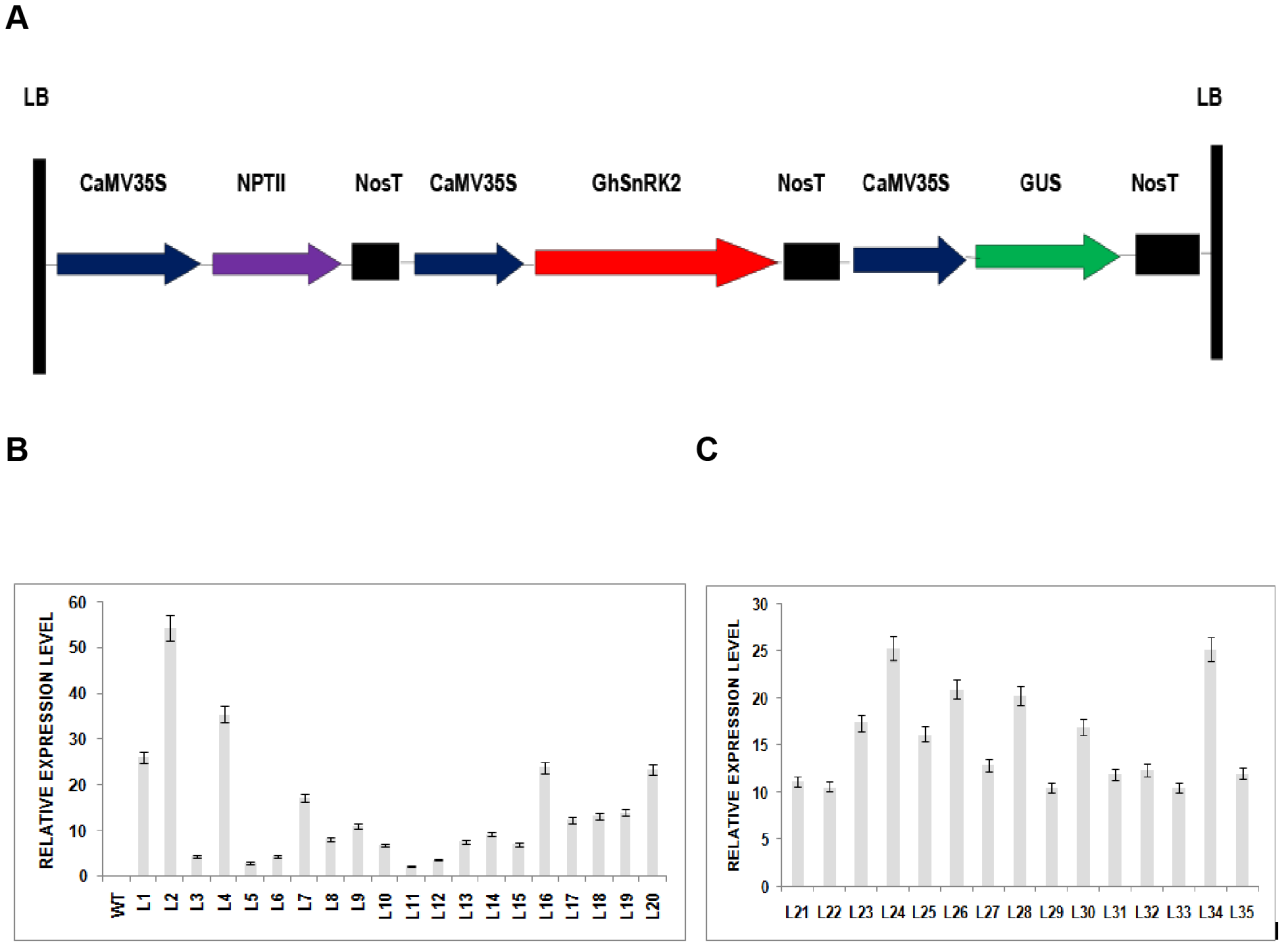
Plant transformation vector and the expression pattern of the *GhSnRK2* gene in the transgenic lines. (A) Schematic representation of the T-DNA region of the binary vector *pCAMBIA2301-GhSnRK2*. (B) Expression pattern of the *GhSnRK2* gene in the transgenic plants. Various upregulated expression patterns of the *GhSnRK2* gene in transgenic lines were detected, as indicated by the vertical axis. The values are presented as the means of three experimental replicates; the error bars indicate the standard deviations. The *AtACT2* gene was used as an internal control for normalization of gene expression.

The schematic representation of the 35S-*GhSnRK2* construct used for *Arabidopsis* transformation (Figure 2A). The full-length *GhSnRK2* cDNA was introduced into the *Arabidopsis* genome via the floral dip method using *Agrobacterium* GV3101; the XbaI–BamHI region of *GhSnRK2* was inserted between the CaMV 35S promoter and the nopaline synthase gene terminator in the pCAMBIA2301 vector to generate the recombinant plasmid *35S-GhSnRK2-NOST*. The neomycin phosphotransferase II (*NPTII*) gene, driven by the nopaline synthase gene promoter (NOSP), was carried by the *pCAMBIA2301* vector for transgenic cell selection in kanamycin-containing LB medium. *GhSnRK2* in transgenic *Arabidopsis* plants was confirmed by PCR analysis. Genomic DNA from the first generation of the plants (T1) was extracted and used as a template for gene-specific primers. Of the 43 plants evaluated, 35 were positive for *GhSnRK2* amplification. No product was formed by the amplification reaction using untransformed plant DNA as the template (Figure S2B). At T1 generation, the ratio of dead to surviving plants was approximately 1∶3 in kanamycin-containing LB medium. However, at T3 generation, all of the plants became homozygous, as their survival rate was 100% in kanamycin-containing LB medium (Figure S2A, Table S1). The values are expressed as the mean germination rate (%) of approximately 200 seeds. qRT-PCR analysis was performed to evaluate the differences in transgene expression between all of the independently generated transgenic lines. Various transgene expression patterns were detected in the transgenic lines overexpressing the *GhSnRK2* gene, but no expression was detected in the WT line, indicating that the *GhSnRK2* genes were successfully introduced into the transformed plants (Figure 2B). The values are presented as the means of three experimental replicates; the error bars indicate the standard deviations. *AtACT2* (gene accession At3G18780) was used as internal control to normalize expression data. Three transgenic lines (L1, L2, and L4) were selected for further investigation based on their seed availability and relative expression pattern.

### Overexpression of *GhSnRK2 in transgenic plants improves their tolerance to drought and cold stresses*

**Figure 3:**
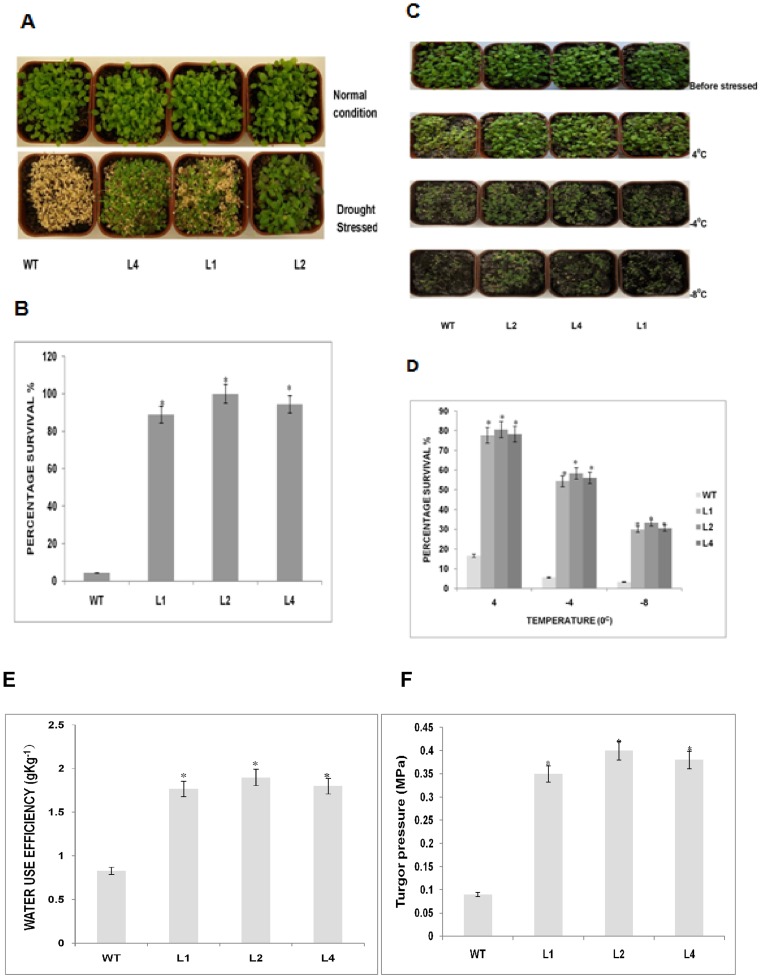
Survival rates of *GhSnRK2* transgenic plants under drought and low temperature stresses. (**A**) WT and *GhSnRK2* transgenic plants before and after drought stress. (**B**) Seedlings were cultured for 2 weeks with constant watering before the withholding of water. After 9 days without water, all of the plants were irrigated, and plant re-growth was scored 4 days later. The plants were scored as survivors if there were healthy green young leaves after re-watering. The survival rate was calculated as the ratio of the number of surviving plants to the total number of treated plants in the flower pot. Asterisk denotes a significant difference (P<0.05). (**C**) WT and *GhSnRK2* transgenic plants before and after low temperature stress. (**D**) Survival rates under low temperature stress conditions. The survival rate after transferring 4-week-old *GhSnRK2* transgenic and WT plants to a low temperature chamber at 4°C, −4°C or −8°C for 10 h, followed by returning the plants to normal growth conditions. Clearly green plants after returning to the normal growth condition were scored as survivors, and plants exhibiting clear signs of wilting were denoted as dead. The mean survival rates of the WT line were compared with those of the transgenic lines using Student's T-test. (E) The water use efficiency of the WT and *GhSnRK2* transgenic plants. Asterisk denotes a significant difference (P<0.05).

To investigate the roles of *GhSnRK2* in stress response pathways, the responses of the three selected transgenic *GhSnRK2*-overexpressing *Arabidopsis* lines to drought and low temperature stresses were analyzed. Constitutive overexpression of the 35S-*GhSnRK2* gene resulted in an increase in the drought tolerance of the transgenic plants. After withholding water for 9 days, some of the WT plants grew slowly, wilted severely and died, whereas few of the *GhSnRK2* transgenic plants wilted. The recovery rate of the transgenic plants was higher than that of the WT plants after re-watering. Indeed, the survival rate was significantly different between the *GhSnRK2* transgenic and wild-type plants: L2 (100%), L4 (94.4%), L1 (88.9%), and WT (4.4%); Student's T-test (n = 3) (p<0.05) (Figure 3A, B). These results suggested that the *GhSnRK2* gene is involved in the response to drought tolerance.

Under low temperature stress conditions, the survival rate of the transgenic lines was significantly higher than that of the WT line. Most of the transgenic plants were intact and recovered from this stress, whereas most WT plants were found to have died and could not recover after transferring them to the normal growth conditions. The survival rates at 4°C were: WT (16.67%), L2 (80.55%), L1 (77.78%), and L4 (78.33%). The survival rates at −4°C were: WT (5.56%), L1 (54.44%), L2 (58.33%), and L4 (56.11%). At −8°C, only 3.3% of the WT plants survived, whereas the survival rates of transgenic lines ranged between 30% and 33.33% after 7 days under normal growth conditions (Figure 3C, D). However, the survival rate under different low temperatures clearly indicates the difference between the transgenic and WT plants, suggesting that the *GhSnRK2* overexpression alleviates low temperature stress in transgenic Arabidopsis. Our findings revealed that the water use efficiency was higher in *GhSnRK2*-overexpressing plants compared to the corresponding WT plants, suggesting that *GhSnRK2*-overexpressing plants exhibit greater photosynthetic potential during drought stress treatment (Figure 3E). Normal turgor pressure which is regulated by the amount of water in the plant's cells is required for healthy growth of a plant and is a powerful determinant of the plant's drought tolerance. We determined the turgor of the *GhSnRK2* transgenic plant to understand the role of turgor pressure in cellular signaling during water deficit condition. Turgor values of *GhSnRK2* transgenic plant was notably higher compared to the corresponding WT plant and this may be attributable to osmotic adjustment, like stomatal closure, and the maintenance of water content which prevent plants from desiccation and turgor loss. Student's T-test (n = 3) (p<0.05) (Figure 3F).

### Biochemical and physiological assays of plants overexpressing the *GhSnRK2* gene

**Figure 4:**
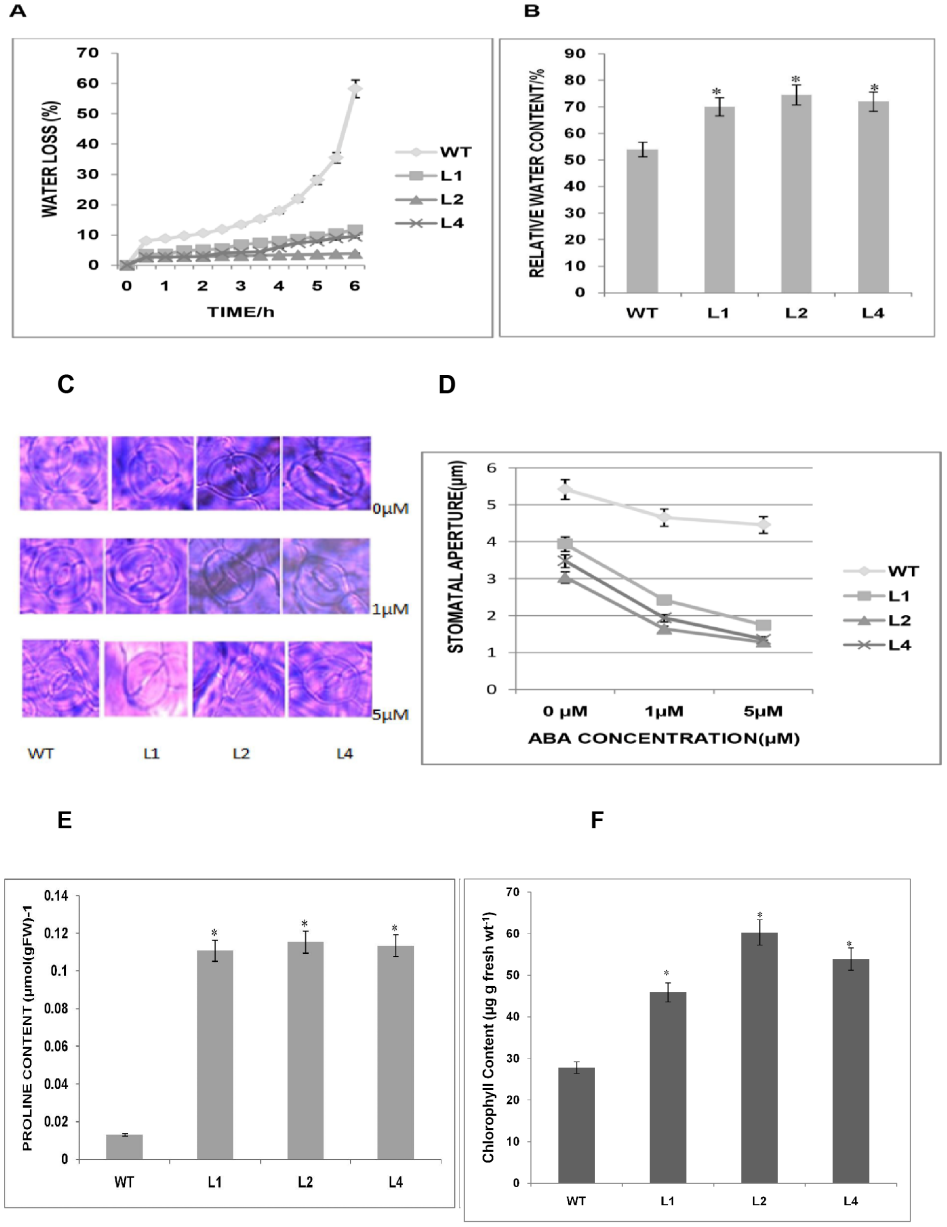
Biochemical and physiological assays of plants overexpressing the *GhSnRK2* gene. (A) Rate of water loss from *GhSnRK2* transgenic plants. Six plants of each transgenic and WT line were analyzed. Three biological replicates produced similar results. Asterisk denotes a significant difference (P<0.05). (B) The RWC of *GhSnRK2* transgenic plants. (C) Stomata aperture size of WT and *GhSnRK2* transgenic plants treated with different concentrations of ABA (D) The stomata apertures were measured after 2 h of treatment with different concentrations of ABA, and the mean values of the WT and transgenic lines at each ABA concentration were compared using Student's T-test (P<0.05). Asterisk denotes a significant difference (P<0.05). (E) Proline accumulation in WT and *GhSnRK2* transgenic plants. The proline content of *GhSnRK2* transgenic lines was consistently higher than that of the WT line. Student's T-test revealed a significant difference (p<0.05) between the transgenic and WT lines (n = 3). (F) The chlorophyll content of the WT and *GhSnRK2* transgenic plants. The mean values were compared using Student's “t-test”. Asterisk denotes a significant difference (P<0.05).

The rate of water loss and the RWC were investigated to further understand the tolerance of *GhSnRK2-*overexpressing plants to water stress via the maintenance of a higher RWC and a reduced rate of water loss. Our results revealed that due to their smaller stomata aperture, the transgenic *Arabidopsis* plants overexpressing the *GhSnRK2* gene lost water more slowly than the WT plant in the same period under normal conditions, which may underlie the capacity of the transgenic plants to maintain a higher leaf RWC and to tolerate water stress conditions; “tstat”>t Critical two-tail (Figure 4A). The *GhSnRK2-*overexpressing plants exhibited a higher RWC than the WT plants, which is most likely due to the reduction in transpiration-mediated water loss. The RWCs in each line were L2(74.56%), L4(72.09%), L1 (70.04%), and WT (53.98%); Student's T-test (n = 3) (p<0.05) (Figure 4B). It is known that during the plant response to abiotic stress, stomata typically close to reduce the rate of water loss due to transpiration. Moreover, abscisic acid (ABA) regulates the stomatal aperture under water-deficient conditions. We found that the stomata apertures of the *GhSnRK2* transgenic plants in response to exogenous ABA treatment were smaller than those of the WT plants, indicating the protective effect of the *GhSnRK2* protein in response to ABA. At 0 µm ABA, the stomata aperture of the WT plants was observed slightly wider than that of the *GhSnRK2* transgenic plants. However, when the concentration of ABA was increased to 1 µm, the aperture size of the transgenic plants was significantly reduced. A similar pattern was detected at 5 µm ABA (n = 5) (Figure 4C, D). Free proline is an osmoprotective molecule that accumulates under stress conditions. Our results revealed that the proline contents of the *GhSnRK2* transgenic lines were significantly higher than those of the WT line, indicating that the transgenic plants accumulate a higher amount of proline than the WT plants. Moreover, the upregulation of Arabidopsis pyrroline-5-carboxylate synthetase 1, a proline biosynthesis gene, in *GhSnRK2*-overexpressing plants suggested that proline production may be improved by *GhSnRK2* via the regulation of proline biosynthesis genes; Student's T-test (n = 3) (p<0.05) (Figure 4E). Greater leaf chlorophyll content at all stages of plant development has been associated with improved transpiration efficiency under drought stress, and this trait may indicate the presence of drought avoidance mechanisms. Based on our results, the chlorophyll content of the *GhSnRK2* transgenic lines was higher than that of the WT line, suggesting that *GhSnRK2*-overexpressing plants exhibit greater photosynthetic potential (p<0.05) (Figure 4F).

### Overexpression of *GhSnRK2* in transgenic plants enhances seedling growth in response to NaCl and exogenous ABA treatment

**Figure 5:**
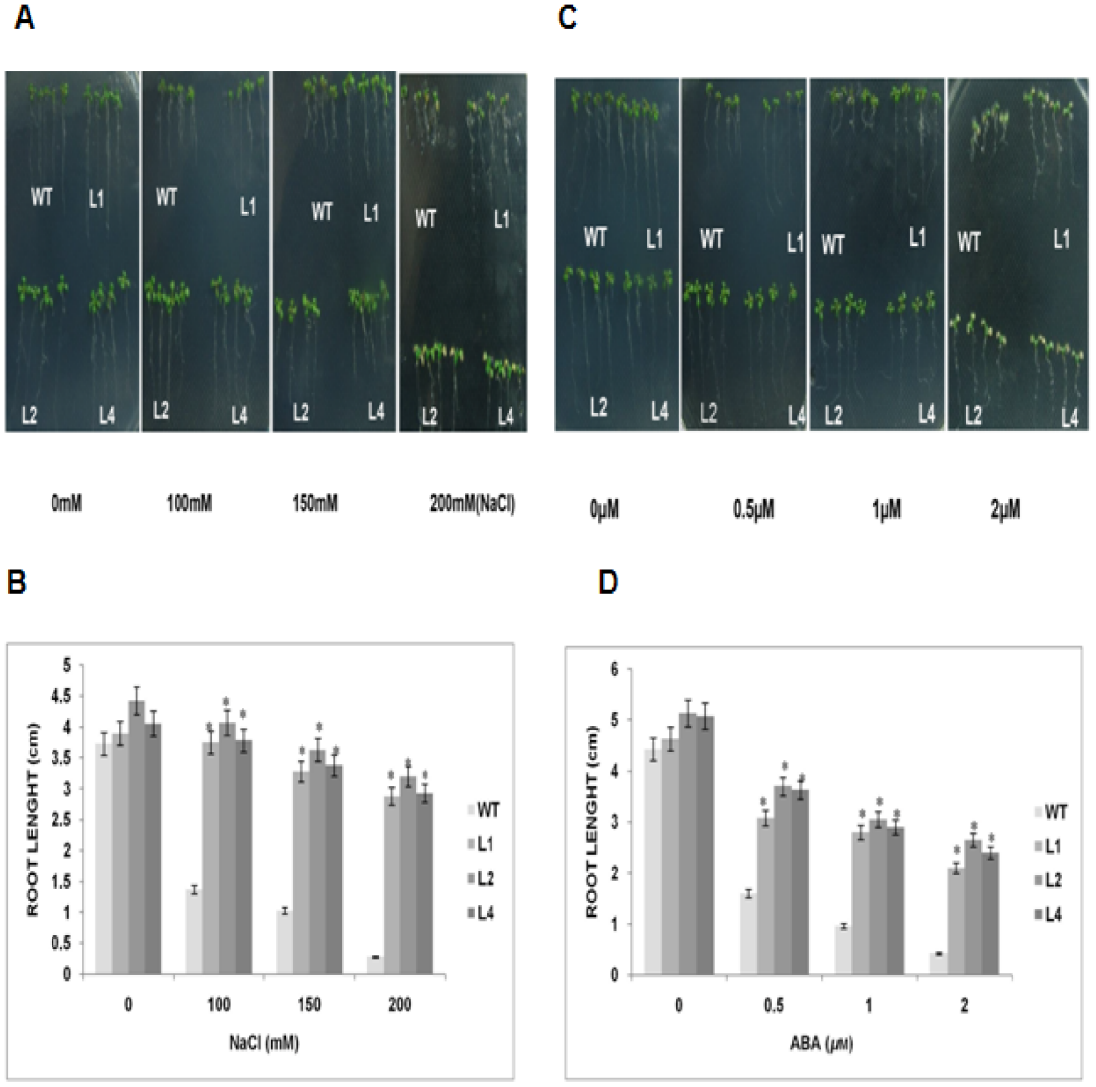
Seedling growth of the WT and *GhSnRK2*-overexpressing lines in response to NaCl and ABA treatment. (A) WT and *GhSnRK2* transgenic plants subjected to different concentrations of NaCl. (B) Approximately one-week-old seedlings were transferred to 1/2MS medium supplemented with different concentrations of NaCl; the root length was measured after 7 days. Each of the three biological replicates consisted of 16 plants. The mean values were compared using Student's T-test (p<0.05). (C) The root growth of the WT and *GhSnRK2* transgenic plants treated with different concentrations of ABA. (D) The plants were cultured vertically on MS-containing medium treated with different concentrations of ABA (0.5 *µ* m, 1 *µ* m, or 2 *µ* m); the root length was measured after 7 days. Each of the three biological replicates consisted of 16 plants. Student's T-test was used to compare the mean values of the WT and transgenic lines. Asterisk denotes a significant difference (P<0.05).

To investigate the effect of *GhSnRK2* overexpression in response to NaCl and exogenous ABA treatment, a root growth experiment was conducted to elucidate the physiological differences between the transgenic and WT plants. The transgenic plants overexpressing *GhSnRK2* exhibited enhanced seedling growth under NaCl and ABA stress treatments, suggesting that *GhSnRK2* may be involved in the oxidative stress response pathway. Seedlings of WT and *GhSnRK2* transgenic lines grew normally in 0 mM NaCl. In 100 mM and 150 mMNaCl, the transgenic seedlings formed longer roots and displayed significantly larger growth than the WT seedlings. When the NaCl concentration was increased to 200 mm, the growth of the WT seedlings was completely inhibited, and the color of these seedlings was found to have turned brownish, whereas the transgenic seedlings remained green and continued to grow, although at a slower rate (n = 3) (Figure 5A, B).

The seedlings from the WT and *GhSnRK2*-overexpressing lines cultured under normal conditions displayed no significant difference. However, when various concentrations of ABA (0.5 *µ* m, 1 *µ* m, or 2 *µ* m) were introduced to the MS medium and the plants were allowed to grow vertically, the root growth of the *GhSnRK2*-overexpressing lines, although slightly inhibited, displayed greater elongation than the WT line, the root growth of which was severely inhibited (n = 3). These results indicated that *GhSnRK2*-overexpressing plants were more tolerant to ABA than the WT plants (Figure 5C, D).

### *GhSnRK2* overexpression in transgenic plants enhances seed germination in response to NaCl and exogenous ABA treatment

**Figure 6:**
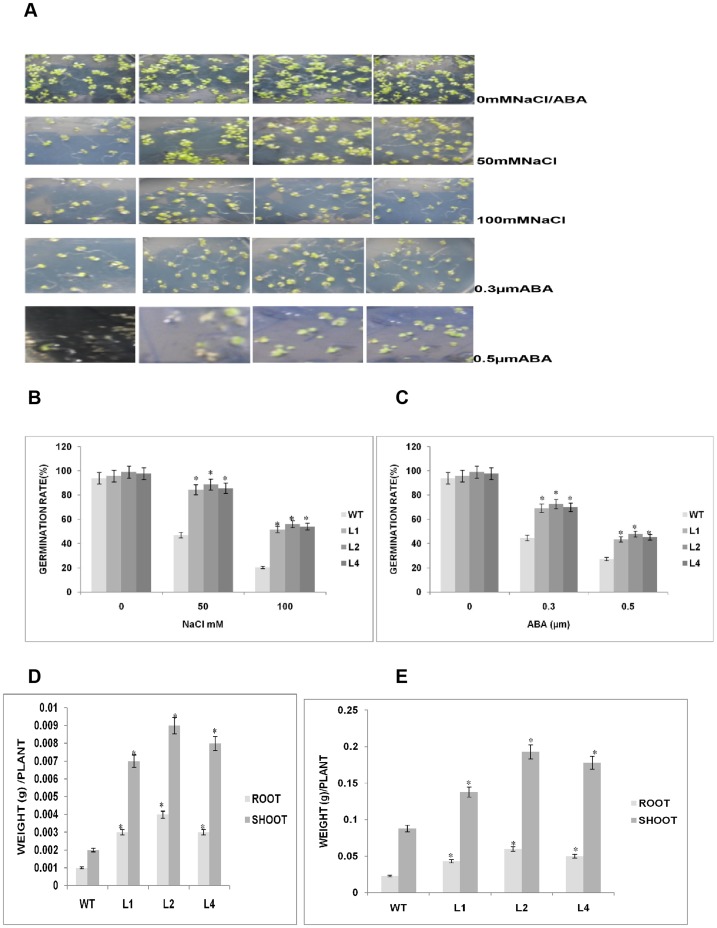
Seed germination of the WT and *GhSnRK2* plants subjected to NaCl and exogenous ABA treatment and biomass accumulation of these plants. (A) Seed germination frequency of the WT and *GhSnRK2* transgenic plants cultured on MS medium supplemented with different concentrations of NaCl (50 mM or 100 mM) or ABA (0 µM, 0.3 µM, or 0.5 µM). (B) The survival rate of the WT and *GhSnRK2* transgenic plants cultured in MS medium containing 50 mM or100 mMNaCl. The mean values were compared using Student's T-test (p<0.05). (C) The germination rate in MS medium supplemented with 0.3 µM or 0.5 µM ABA. The values are presented as the mean germination rates (%) of approximately 200 seeds. Asterisk denotes a significant difference (P<0.05). (D) Biomass accumulation of the *GhSnRK2* transgenic and WT plants. For dry weight biomass, the dry weight in the roots and shoots was recorded after drying in an oven to a constant weight at 70°C for 48 h. (E) Fresh weight biomass of *GhSnRK2* transgenic plants and corresponding WT plants. The fresh weight of the roots and shoots was measured immediately after harvesting. Each of the three biological replicates consisted of 12 plants. Student's T-test was performed. Asterisk denotes a significant difference (P<0.05).

We investigated the germination of *GhSnRK2-*overexpressing plants under exogenous ABA and salt stresses to determine whether *GhSnRK2* is involved in stress response pathways. The germination rates of WT and *GhSnRK2*-overexpressing seeds were similar at 0 mM NaCl and ABA. However, when different concentrations of ABA or NaCl were introduced, the germination of the WT and *GhSnRK2*-overexpressing seeds was inhibited. When 50 mM NaCl was introduced to the “MS” medium, the germination rate was 47% for the WT line and 85%, 89%, and 86% for the L1, L2, and L4 lines, respectively. At100 mM NaCl, the germination rates were: WT (20.5%), L1(52%), L2 (56%), and L4 (54%)(Figure 6A, B). These results suggested that the *GhSnRK2*-overexpressing plants were more tolerant to salt stress than the corresponding WT plants. ABA plays a prominent role in the regulation of germinating and post-germinating growth arrest and mediates the adaptation of the plant to stress. To investigate this hypothesis, we measured the response of *GhSnRK2*-overexpressing plants to different concentrations of ABA during the germination stage. At 0.3 µm ABA the seed germination rates were WT (45%), L1 (69%), L2 (73%), and L4 (70%), respectively. When the concentration of ABA was increased to 0.5 µm, the germination rate of the WT line was clearly reduced (27%), whereas the germination rate of the transgenic lines ranged from 43% to 48% (Figure 6A, C). In summary, our findings revealed that the *GhSnRK2*-overexpressing mutants were more tolerant to NaCl and exogenous ABA stresses than the corresponding WT plants. Student's t-test was used to compare the mean germination rates. Asterisks denote a significant difference compared to the control (*P*≤0.05).

We further investigated the biomass accumulation of *GhSnRK2* transgenic and WT plants. The transgenic plants exhibited highly significant increases in both the fresh and dry weight biomasses compared with the corresponding WT plants (Figure 6D, E). These increases were all statistically significant; suggesting that overexpression of the *GhSnRk2* gene increases biomass accumulation in plants. The increased biomass accumulation may be attributable to the increased chlorophyll content in *GhSnRK2* overexpressing plant, as chlorophyll in plant cells carries out the bulk of energy fixation in the process of photosynthesis and is probably the most-often used estimator of plant biomass.

### Expression analysis of stress-responsive marker genes

**Figure 7:**
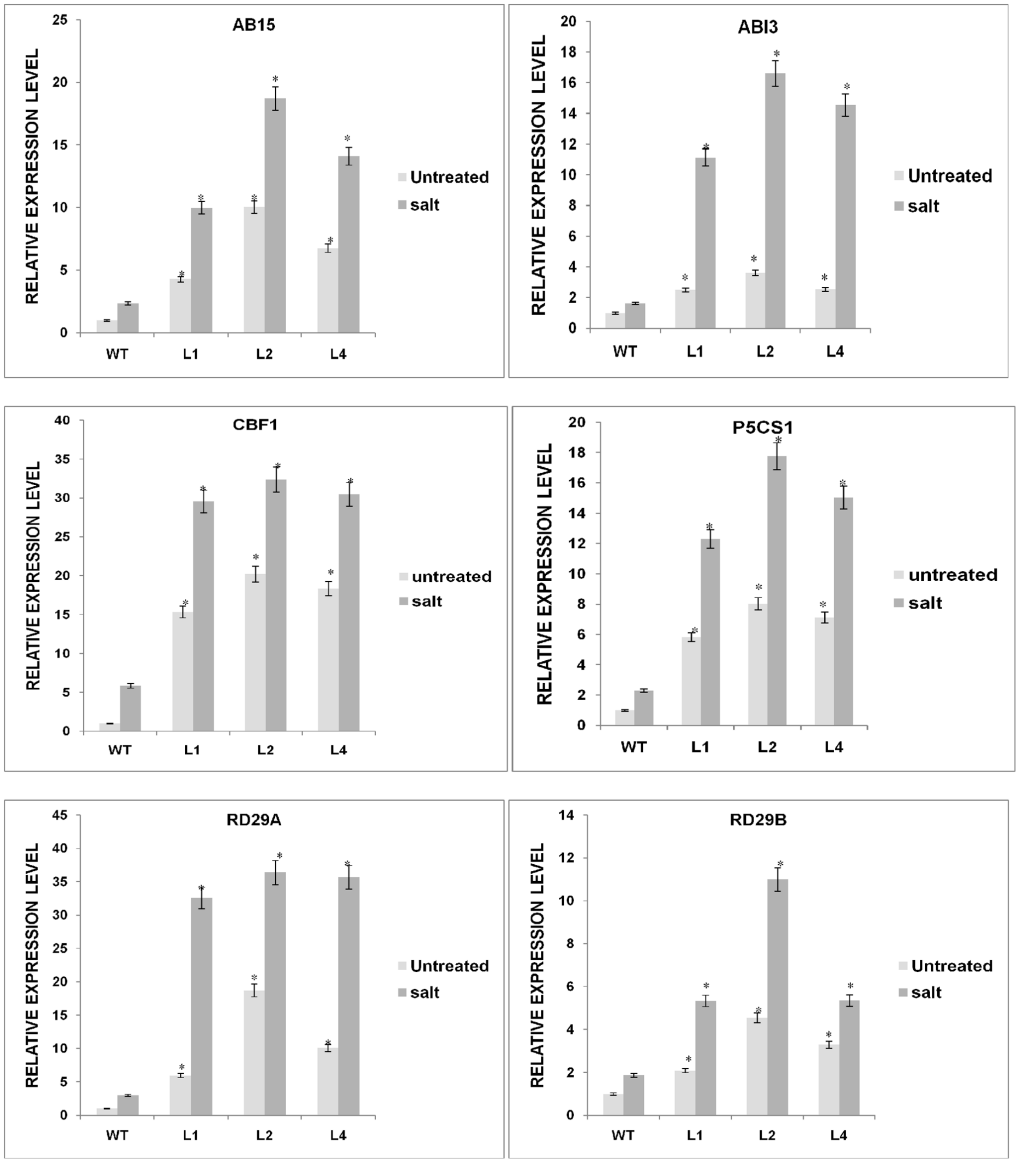
Expression analysis of stress-responsive marker genes. The relative transcript levels of the stress-responsive genes *AtABI5, AtABI3, AtP5CS1, AtRD29A, AtCBF1*, and *AtRD29B* in the *GhSnRK2*-overexpressing and WT lines. qRTPCR was performed for gene expression analysis. The vertical axis displays the expression pattern. Three biological replicates produced similar results.

To further elucidate the biological function and molecular mechanisms of the *GhSnRK2* gene, we determined the transcript levels of several stress-associated genes in *GhSnRK2*-overexpressing lines. Seedlings from 10-day-old *GhSnRK2* transgenic and WT *Arabidopsis* lines cultured in MS medium were in the presence or absence of NaCl (250 mM) for 2 h. qRT-PCR was performed as described in the Materials and Methods section. Our findings revealed that *GhSnRK2* regulates the expression levels of ABA and stress-responsive marker genes, suggesting that *GhSnRK2* may positively affect ABA signaling and plant stress responses. The expression levels of C-repeat binding factor-1 (*AtCBF1*; accession: ABV27087), delta-1-pyrroline-5-carboxylatesynthase-1 (*AtP5CS1*; accession:NP001189715), protein abscisic acid-insensitive-5 (*AtABI5*; accession:NP565840), protein abscisic acid-insensitive-3 (*AtABI3*; accession:CAA48241), desiccation-responsive genes (*Atrd29B*; accession: BAA02375 and *Atrd29A*; accession:BAA02376) in the *GhSnRK2-*overexpressing lines were significantly higher than those of the WT lines (p≤0.05), revealing that *GhSnRK2* is actively involved in stress signaling pathways (Figure 7). Three experimental replicates produced similar results (n = 3); Student's T-test.

### VIGS efficiency and the transcript level of *GhSnRK2* in gene silenced plants

**Figure 8:**
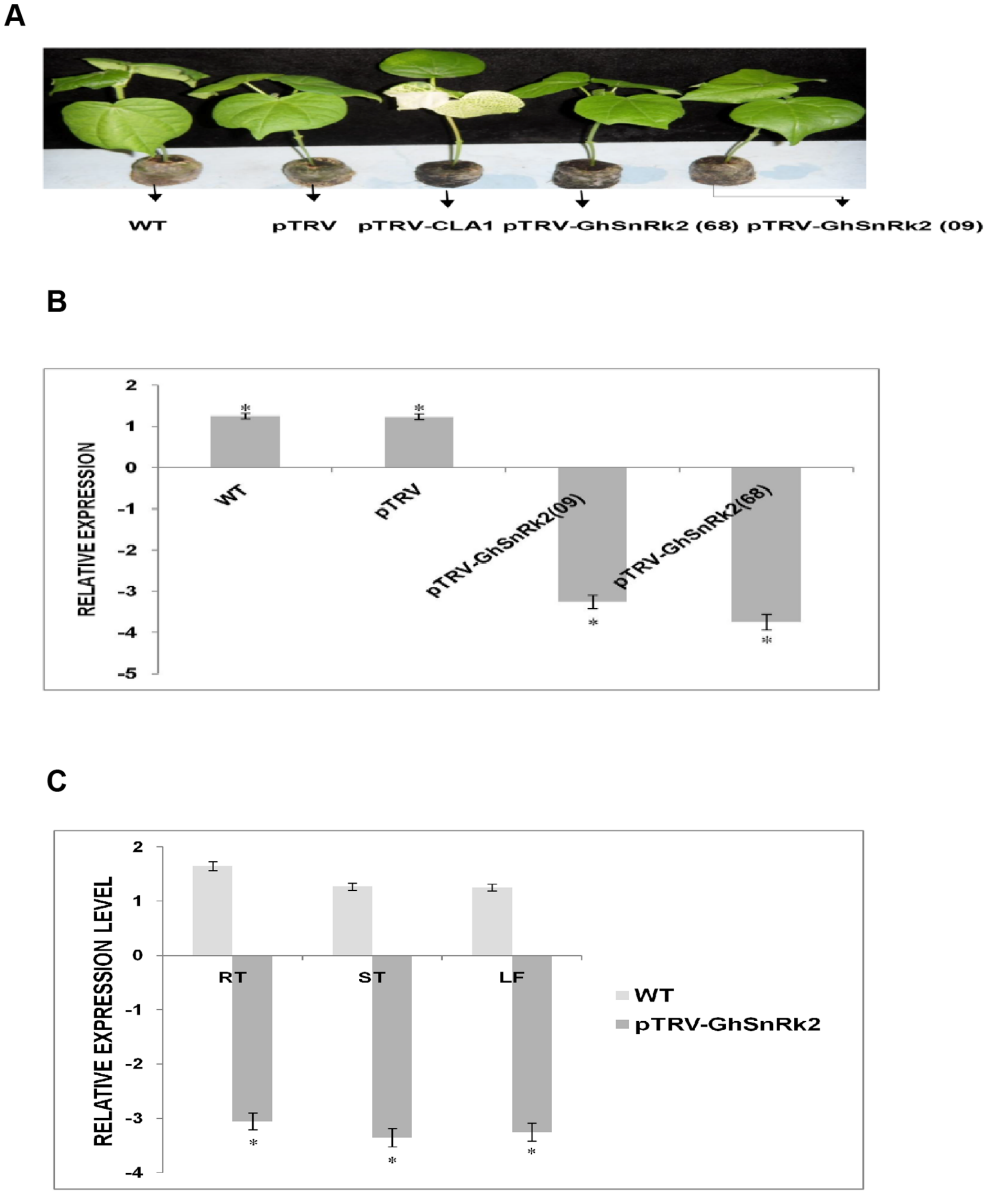
Silencing efficiency and transcript level of the *GhSnRK2* gene silenced plants. (A) Phenotype of gene silenced and non-silenced plants; 14 dpi. Wild-type (WT), negative control Empty vector (pTRV), positive control (pTRV-GrCLA1), gene silenced CRI409 cotton cultivars [pTRV-GhSnRk2 (09)], and gene silenced CRI99668 cotton cultivars [pTRV-*GhSnRK2* (68)]. (B) The silencing efficiency as determined by the expression pattern of WT, vector control (pTRV) and *GhSnRK2* gene silenced plants based on qRT-PCR. (C) The distribution of the TRV- construct in the gene silenced plants. Samples from the root, the stem and the leaves of gene silenced and non-silenced plants were analyzed via qRT-PCR. The values are presented as the means of three biological replicates. Asterisk denotes a significant difference (P<0.05).

We employed the VIGS technique to further dissect the function of the *GhSnRK2* gene in transgenic cotton. The TRV VIGS vectors were modified based on a pTRV1 containing RNA-dependent RNA polymerase (RdRp), movement protein (MP), a 16 kDa cysteine-rich protein (16K), CaMV 35S promoters (2X35S) and a NOS terminator (NOSt) T-DNA vector. pTRV2 contains the coat protein (CP), multiple cloning sites (MCSs), CaMV 35S promoters (2X35S) and a NOS terminator (NOSt) T-DNA vector. Both vectors contain Rz, which is designated as a self-cleaving ribozyme, LB (left border) and RB (right border) of the T-DNA (Figure S1B). After inoculating *Agrobacterium* strains containing *GhSnRK2* derivatives (PYL156: (pTRV-RNA2)-*GhSnRk2,* PYL192:(pTRV-RNA1), PYL156:(pTRV-RNA2)-*GhCLA1 (*positive control), or PYL156:(pTRV-RNA2) vector) into *Gossypium hirsutum* leaves, the phenotype of both silenced and non-silenced plants was monitored daily for gene silencing efficiency. Loss of normal green coloration in plant leaves and albino phenotype was detected in PYL156:(pTRV-RNA2)-*GrCLA1* silenced plants, which served as the positive control indicating gene silencing efficiency. The *GrCLA1* gene represents the ideal visual marker for gene silencing efficiency due to its involvement in chloroplast development, as its loss of function results in an albino phenotype in true leaves (Figure 8A). The transcript levels of *GhSnRK2* in gene silenced and non-silenced plants were analyzed via qRT-PCR. The transcript level of *GhSnRK2* in the gene silenced two plant cultivars were downregulated compared to the non-silence plants inoculated with empty vector and WT plants, which exhibited a higher expression level. The downregulation of *GhSnRK2* indicated that the gene was successfully knocked down in the silenced plants (Figure 8B). qRT-PCR analysis of the distribution of the TRV-construct in the silenced plants revealed that its infection results in gene-specific transcript degradation, that pTRV-*GhSnRK2* functions in diverse tissues, and that pTRV-*GhSnRK2* is abundantly expressed in the root. The roots of gene silenced plants exhibited further down regulated expression levels compared to the stems and the leaves. The TRV-construct displays increased infectivity and meristem invasion, both of which are key requirements for efficient VIGS-based functional characterization of genes in root tissues (Figure 8C).

### Physiological assay and responses of *GhSnRK2* gene silenced plants to various stresses

**Figure 9:**
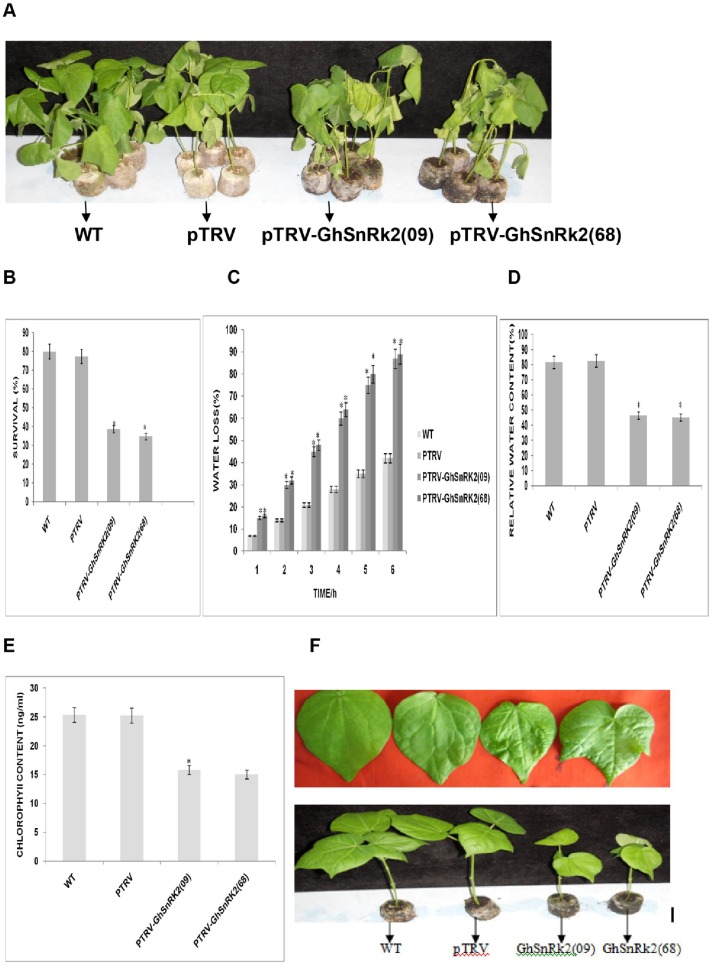
Physiological assay of *GhSnRK2* gene silenced plants. (A) Phenotype of drought stressed plants. Water was withheld from *GhSnRK2* gene silenced and non-silenced plants for 5 days approximately two weeks post-inoculation. Each of the ten treated groups consisted of five plants. A photograph of each group representative was captured. (B) The survival rate was determined by withholding water from *GhSnRK2* gene silenced and non-silenced plants for 5 days approximately two weeks post-inoculation, and the survival rate was recorded as the percentage of plants that survived after re-watering for 3 days. (C) The relative water loss was determined two weeks after inoculation. The reduction in the fresh weight from the initial weight was determined at the indicated time and represented as the percentage of water loss. The experiment was replicated three times. (D) The mean RWC was analyzed after immersing the fresh detached leaves in distilled water for 4 h and oven drying at 80°C for 48 h. The values are presented as the means of three biological replicates. Asterisk denotes a significant difference (P<0.05). (E) The change in the chlorophyll content of *GhSnRK2* gene silenced and non-silenced plants under salt stress. (F) The phenotype of *GhSnRK2* gene silenced and non-silenced plants under salt stress.

To further elucidate the function of the *GhSnRK2* gene in stress tolerance, we investigated the response of *GhSnRK2*gene silenced plants to various stresses. Our findings validated the importance of this technique for stress tolerance studies. Under water-deficient conditions, the silenced plants wilted and drooped, regardless of the cultivar (Figure 9A). The phenotype of the non-silenced plants inoculated with empty vector (TRV) was indistinguishable from that of the WT plants. The two cotton cultivars (CRI99668 and CRI409) inoculated with the target gene displayed similar phenotypes and symptoms. The WT and control vector-treated plant survival rates were 80% and 77.3%, respectively, whereas the survival rate of the two gene silenced cotton cultivars (CRI99668 and CRI409) were 34.66% and 38.67%, respectively (Figure 9B). Student's T-test revealed a significant difference (p<0.05) between the survival rate of the gene silenced and non-silenced plants. The rate of water loss from the detached leaves was determined in both the gene silenced and non-silenced plants (Figure 9C). After monitoring the water loss for 6-h, we found that the *GhSnRK2* gene silenced plants inoculated lost more water than the non-silenced plants inoculated with empty vector and the WT plants. The rate of water loss was slightly higher in the CRI99668 cultivars than in the CRI409 cultivars. Under similar conditions, the gene silenced plants, regardless of the cultivar, exhibited a substantially reduced water content compared with the non-silenced plants inoculated with empty vector and the WT plants (Figure 9D). Oxidative stress, including that mediated by salinity, alters the physiological and morphological responses of plants. To investigate the effect of salt stress on *GhSnRK2* gene silenced plants, the gene silenced and non-silenced plants were treated with 150 mM NaCl for 7 days, and the effect of NaCl was measured following this stress. Our findings revealed that the chlorophyll content of the non-silenced plants was higher than that of the gene silenced plants, indicating that the *GhSnRK2* gene may be involved in the oxidative stress response (Figure 9E). Growth retardation was detected in the *GhSnRK2* silenced plants, and the effect of salt accumulation in the plant cells was detected as elevated blisters, which were visibly detectable on the leaf surface of *GhSnRK2* gene silenced plants (Figure 9F).

## Discussion

The ability of plants to withstand water shortage while sustaining proper physiological activities can be associated with drought tolerance. In this study, we generated a gene construct containing *GhSnRK2* driven by the CaMV 35S promoter and functionally characterized it via gene overexpression in Arabidopsis. We further elucidated the function of this gene in transgenic cotton plants using VIGS techniques. We found that the amino acid sequence of *GhSnRK2* is highly similar to that of other *SnRK2* proteins, and a neighbor-joining tree developed based on an alignment of the complete protein sequences revealed that *GhSnRK2* clustered with known stress-related genes from other plants. The conserved motif detected demonstrated that *GhSnRK2* is a functional protein. The conserved domain (CD) region in these proteins plays a crucial role in protein interactions, DNA binding, enzyme activity, and other important cellular processes. Furthermore, we found that *GhSnRK2* was localized to the cytoplasm and the nucleus, suggesting that *GhSnRK2* might perform diverse functions in cotton plants. Our findings revealed that *GhSnRK2* is abundantly expressed in the root and is widely distributed throughout plant tissues, suggesting that it may function in diverse tissues. Transgenic *Arabidopsis* plants overexpressing *GhSnRK2* exhibited enhanced tolerance to various abiotic stresses. Consistent with previous studies, *SnRK2* genes have been implicated in the response to multi-environmental stresses [16], [30], [31]. The improved drought tolerance of plants overexpressing the *GhSnRK2* gene may be due to the abundance of gene expression in roots. In addition, protein kinases that are specifically found in roots, such as *SnRK2C*, may perform certain important functions in root tissues as a sensor of water and nutrients in soil. The root tip plays a vital role in the response to water and nutrient detection via appropriate signal transduction [32]. Turgor regulation of the *GhSnRK2* transgenic plant under water deficit condition may be attributable to the dynamic process of cell wall adjustment of the transgenic plant due to their enhanced drought-tolerant. Plants can maintain turgor by solute accumulation, i.e. by osmotic adjustment, and possibly by elastic adjustment of their cell walls [33].

Based on our results, the transgenic plants overexpressing *GhSnRK2* exhibited enhanced low temperature tolerance compared to WT plants. This result may be attributable to the resistance of the membrane system of the transgenic plant cells to cold stress. These findings corroborated the results of [30], who reported enhanced multi-stress tolerance in *Arabidopsis* plants overexpressing *TaSnRK2.8*. Numerous studies have demonstrated that the membrane systems of the cell are the primary site of low temperature contusion in plants [34], [35]. Indeed, it is well confirmed that low temperature-mediated membrane injury occurs primarily as a result of acute dehydration associated with low temperature [35], [36].

Our findings revealed the accumulation of compatible osmolytes, such as free proline, in the *GhSnRK2*-overexpressing plants; therefore, we speculate that the *GhSnRK2* transgenic plants exhibit increased stress tolerance by regulating downstream gene expression and accumulating a larger amount of compatible osmolytes, which may account for the increase in stress tolerance of *GhSnRK2* plants. Proline is an osmoprotective molecule that accumulates in response to water stress and salinity [37], [38]. Proline is considered as a typical physiological parameter for evaluating abiotic stress tolerance and resistance in plants. Many plants reduce their cellular osmotic potential via the accumulation of intracellular organic osmolytes, such as proline, to maintain a stable intracellular environment when subjected to external environmental stresses [39], [40], [41].

We detected decreased stomatal apertures in the *GhSnRK2* transgenic plants compared with the corresponding WT plants. When exposed to adverse environmental conditions, the stomata must close for the plant to survive [42]. Stomatal closure reduces water loss, particularly in plants that have been exposed to water stress conditions caused by high solute concentrations in the nutrient medium, such that minimizing the rate of water loss due to transpiration reduces the accumulation of toxic ions [43]. The reduced rate of water loss from *GhSnRK2* transgenic plants may be due to the decrease in the size of the stomata aperture. ABA is involved in stomata movement, enhanced drought tolerance and plant osmoregulation. Under water stress conditions, ABA is released into leaves, inducing the release of potassium salts from the guard cells, resulting in stomatal closure. Plant guard cells modulate the opening and closure of the stomata in response to phytohormones and various environmental signals, such as light and temperature, thereby regulating gas exchange for photosynthesis and water status via transpiration [44]. The *SnRK2* gene *SRK2E*, or *SnRK2.6*, plays a significant role in stomatal closure in *Arabidopsis* leaves [45], [46].

The *GhSnRK2* gene was upregulated after treatment with 10% PEG, which may suggest that the *GhSnRK2* gene sufficiently confers drought tolerance, as one strategy that can be employed to mimic the effect of drought on plants is treatment with PEG. PEG was quite commonly used in physiological experiments to induce controlled drought stress.

We speculate that the *GhSnRK2*-overexpressing plants were more tolerant to NaCl than the WT plants, which may be due to the improved accumulation of Na+ in the vacuoles, increased sequestration of Na+ into the vacuoles, improved cellular Na+ exclusion, or their greater capacity for vacuolar osmoregulation. High salt can result in oxidative damage to cell membranes. The ability to avert such damage is consistent with the degree of tolerance exhibited by the plant [47]. Under salt stress, this damage is less extensive in transgenic plants overexpressing the *GhSnRK2* gene than in WT plants because *GhSnRK2* overexpression can tolerate the accumulation of Na+ in the vacuoles, thus preventing the toxicity of excess Na+. Hypersensitivity to ABA treatment during seed germination and early seedling development is typically followed by improved drought tolerance [48], [49]. Our finding reveals that the transgenic plants overexpressing *GhSnRK2* exhibited enhanced seedling growth under ABA stress treatment. This may be attributable to ABA-independent signaling pathway involving SnRK2 in root growth. This finding reveals the complexity in ABA-signaling, as different signaling components may function in different cells or tissues in ABA signal transduction. *SnRK2.2* and *SnRK2.3* double mutant showed strong ABA-insensitive phenotypes in seed germination and root growth inhibition.[50]. The decreased stomatal apertures detected in the *GhSnRK2* transgenic plants may be attributable to the role of *SnRK2* in the regulation of ABA-induced activation of plasma membrane anion channels in guard cells and concomitant response to their closure. These results suggest that *SnRK2* functions in ABA signaling of stomatal closure. *SRK2E* knockout mutant lost the ABA-dependent stomatal closure. SRK2E affects ABA-signaling in stomatal closure, but not in germination stage [51]. ABA and PYR/PYLs inhibit protein phosphatase2C (PP2C), which in turn relieves the repression of positive factors, such as SnRK2s [52].

We analyzed the expression of the stress-inducible marker genes *RD29A, RD29B, P5CS1, ABI3, CBF1*, and *ABI5* in *GhSnRK2*-overexpressing plants, and our results revealed increases in the transcript levels of these marker genes. Consistent with a previous study, the upregulation of stress response genes, such as *P5CS1*, can contribute to enhanced salt stress tolerance in plants [53]. *ABI3* and *ABI5* activation is necessary to sustain the germination of seedlings during intense drought stress [54]. *ABI5* and *ABI3* regulate numerous ABA responses, such as osmotic water permeability of the plasma membrane, stomatal closure, drought-induced resistance, germination, and inhibition of vegetative growth. We speculated that *GhSnRK2* functions in the transcriptional regulation of ABA-inducible genes in seedlings, suggesting that *SnRK2* protein kinases are involved in several processes of ABA signal transduction, including transcriptional as well as post-transcriptional regulation pathways. Therefore, we suggest that stress signaling pathways may be involved in *GhSnRK2*-mediated stress tolerance.

Our findings revealed the downregulation of *GhSnRK2* in gene silenced plants, indicating that this gene was knocked down in the gene silenced plants. TRV spread vigorously throughout the entire plant, including meristem tissue, and the symptoms induced by TRV are not severe compared with those induced by other viruses [55]. The albino phenotype observed in the *GhCLA1*gene silenced plants in this study is an indicator of effective VIGS function in inoculated plants, which is consistent with previous findings [56]. The results of this study revealed that *GhSnRK2* gene silencing in *Gossypium hirsutum* greatly reduced its tolerance to drought stress, corroborating the findings of [57], who reported that silencing of the function of the *SpMPK1*, *SpMPK2*, and *SpMPK3* genes in tomato plants alleviates their tolerance to drought stress. The decrease in the chlorophyll content of silenced plants in response to salt stress indicates that *GhSnRK2* may be involved in oxidative stress. High-concentration salt stress inhibits plant biochemical processes. Decreased chlorophyll content in salt-stressed pumpkin plants was detected by [58]. The stunted growth observed in salt-stressed *GhSnRK2* gene silenced plants corroborate the findings of [59], who reported that the typical effect of salt stress in plants is growth retardation as a result of cell elongation inhibition.

## Conclusions

Despite the significant innovations that have been made in elucidating the genetic mechanisms underlying drought tolerance, considerable challenges remain. The present study revealed that the genetic manipulation of the *GhSnRK2 g*ene from cotton using a transgenic technique results in enhanced drought and cold stress tolerance in plants. Thus, *GhSnRK2* is hypothesized to participate in the stress signaling pathway, and therefore, overexpression of *GhSnRK2* may alleviate abiotic stress by regulating stress-responsive genes. Moreover, the findings in this study have helped to elucidate that *GhSnRK2* enhances stress tolerance in plants by affecting various stress-related pathways. Thus, the *GhSnRK2 g*ene represents a candidate gene for future research of abiotic stress signaling pathways and the genetic modification of novel *Gossypium hirsutum* varieties.

## Supporting Information

Figure S1 **Multiple sequence alignment of** ***GhSnRK2*** **and closely related** ***SnRK2s*** **from other plants species and VIGS construct.** (A) Alignment of *GhSnRK2* and closely related *SnRK2s*. The relatively conserved motif is underlined. The deduced amino acid sequence displays relatively high homology with the monocot *SnRK2* family members *Oryza sativa* (*RK1*), ABB89146 and with the dicot species *AtSnRK2.10*, AEE33751.1. (B) Virus-induced gene silencing construct. The TRV VIGS vectors were modified based on a pTRV1 containing RNA-dependent RNA polymerase (RdRp), movement protein (MP), a 16 kDa cysteine-rich protein (16K), CaMV 35S promoters (2X35S) and a NOS terminator (NOSt) T-DNA vector. pTRV2 contains the coat protein (CP), multiple cloning sites (MCSs), CaMV 35S promoters (2X35S) and a NOS terminator (NOSt) T-DNA vector. Both vectors contain Rz, which is designated as a self-cleaving ribozyme, LB (left border) and RB (right border) of the T-DNA.
(TIF)

Figure S2 **Survival rate in kanamycin-containing medium and PCR confirmation of** ***GhSnRK2*** **gene expression in transgenic Arabidopsis.** (A) The survival rate of WT and *GhSnRK2* transgenic plants in MS medium supplemented with the antibiotic kanamycin. Photograph of a representative plant was captured after 9 days of germination in kanamycin-containing medium. (B) Confirmation of *GhSnRK2* gene in transgenic Arabidopsis. Genomic DNA from the first generation of the plants (T1) was extracted and used as a template for gene-specific primers. Lane 1: DNA molecular marker III standard; lane 2: negative control.
(TIF)

Table S1 **Survival (%) of** ***GhSnRK2*** **transgenic plants in kanamycin-containing medium.** At T1 generation, the ratio of dead to surviving plant was approximately 1∶3 in kanamycin-containing LB medium. The survival rate at T3 generation was 100% in kanamycin-containing LB medium. The values are expressed as the mean germination rate (%) of approximately 200 seeds.
(DOCX)

Table S2 **The lists of primers sequences used in this study.**
(DOCX)

## References

[1] Genoud T, Metraux JP (1999) Crosstalk in plant cell signaling: structure and function of the genetic network. Trends in Plant Science 4: 503–507. 10562736 10.1016/s1360-1385(99)01498-3

[2] Kumar N, Bhatt RP (2006) Transgenics: An emerging approach for cold tolerance to enhance vegetables production in high altitude areas. Indian J. Crop Sci 1: 8–12.

[3] Shinozaki K, Yamaguchi-Shinozaki K (2000) Molecular responses to dehydration and low temperature: differences and cross-talk between two stress signaling pathways. Curr Opin Plant Biol 3: 217–223. 10837265

[4] Guo-Tao H, Shi-Liang M, Li-Ping B, Li Z, Hui M, et al (2000) The role of root border cells in plant defense. Trends Plant Sci 5: 128–133. 10707079 10.1016/s1360-1385(00)01556-9

[5] Cho K, Agrawal GK, Jwa NS, Kubo A, Rakwal R (2009) Rice OsSIPK and its orthologs: a “central master switch” for stress responses. Biochem Biophys Res Commun 379: 649–653. 19116133 10.1016/j.bbrc.2008.12.107

[6] Mishra NS, Tuteja R, Tuteja N (2006) Signaling through MAP kinase networks in plants. Arch Biochem Biophys 452: 55–68. 16806044 10.1016/j.abb.2006.05.001

[7] Pitzschke A, Schikora A, Hirt H (2009) MAPK cascade signalling networks in plant defence. Curr Opin Plant Biol 12: 421–426. 19608449 10.1016/j.pbi.2009.06.008

[8] Rodriguez MC, Petersen M, Mundy J (2010) Mitogen-activated protein kinase signaling in plants. Annu Rev Plant Biol 61: 621–649. 20441529 10.1146/annurev-arplant-042809-112252

[9] Jonak C, Hirt H (2002) Glycogen synthase kinase 3/SHAGGY-like kinases in plants: an emerging family with novel functions. Trends Plant Sci 7: 457–461. 12399181 10.1016/s1360-1385(02)02331-2

[10] Koh S, Lee SC, Kim MK, Koh JH, Lee S, et al (2007) T-DNA tagged knockout mutation of rice OsGSK1, an orthologue of Arabidopsis BIN2, with enhanced tolerance to various abiotic stresses. Plant Mol Biol 65: 453–466. 17690841 10.1007/s11103-007-9213-4

[11] Mahfouz MM, Kim S, Delauney AJ, Verma DP (2006) Arabidopsis TARGET OF RAPAMYCIN interacts with RAPTOR, which regulates the activity of S6 kinase in response to osmotic stress signals. Plant Cell 18: 477–490. 16377759 10.1105/tpc.105.035931 PMC1356553

[12] Das R, Pandey GK (2010) Expressional analysis and role of calcium regulated kinases in abiotic stress signaling. Curr Genomics 11: 2–13. 20808518 10.2174/138920210790217981 PMC2851112

[13] Hrabak EM, Chan CWM, Gribskov M, Harper JF, Choi JH, et al (2003) The Arabidopsis CDPK-SnRK superfamily of protein kinases. Plant Physiol 132: 666–680. 12805596 10.1104/pp.102.011999 PMC167006

[14] Wurzinger B, Mair A, Pfister B, Teige M (2011) Cross-talk of calcium-dependent protein kinase and MAP kinase signaling. Plant Signal Behav 6: 8–12. 21248475 10.4161/psb.6.1.14012 PMC3121996

[15] Coello P, Hirano E, Hey SJ, Muttucumaru N, Martinez-Barajas E, et al (2012) Evidence that abscisic acid promotes degradation of SNF1-related protein kinase (SnRK) 1 in wheat and activation of a putative calcium-dependent SnRK2. J Exp Bot 63: 913–924. 21994172 10.1093/jxb/err320 PMC3254688

[16] Umezawa T, Yoshida R, Maruyama K, Yamaguchi-Shinozaki K, Shinozaki K (2004) SRK2C, a SNF1-related protein kinase 2,improves drought tolerance by controlling stress-responsive gene expression in Arabidopsis thaliana. Proc Natl Acad Sci. USA 101: 17306–173011. 15561775 10.1073/pnas.0407758101 PMC535404

[17] Hardie DG (1999) Plant protein serine/threonine kinases: classification and functions. Annu Rev Plant Physiol Plant Mol Biol 50: 97–131. 15012205 10.1146/annurev.arplant.50.1.97

[18] Mikołajczyk M, Awotunde OS, Muszyńska G, Klessi DF, Dobrowolska G (2000) Osmotic stress induces rapid activation of a salicylic acid-induced protein kinase and a homolog of protein kinase ASK1 in tobacco cells. Plant cell 12: 165–178. 10634915 PMC149182

[19] Wang X, Xu W, Xu Y, Chong K, Xu Z, et al (2004) Wheat RAN1, a nuclear small G protein, is involved in regulation of cell division in yeast. Plant Sci 167: 1183–1190.

[20] Clough SJ, Bent AF (1998) Floral dip: a simplified method for Agrobacterium-mediated transformation of Arabidopsis thaliana. Plant J 16(6): 735–743. 10069079 10.1046/j.1365-313x.1998.00343.x

[21] Tyree MT, Hammel HT (1972) The measurement of the turgor pressure and the water relations of plants by the pressure-bomb technique. J Exp Bot 23: 267–282.

[22] Duan J, Zhang M, Zhang H, Xiong H, Liu P, et al (2012) OsMIOX, a myoinositol oxygenase gene, improves drought tolerance through scavenging of reactive oxygen species in rice (Oryza sativa L.). Plant Sci 196: 143–151. 23017909 10.1016/j.plantsci.2012.08.003

[23] Parida AK, Dasgaonkar VS, Phalak MS, Umalkar GV, Aurangabadkar LP (2007) Alterations in photosynthetic pigments, protein, and osmotic components in cotton genotypes subjected to short-term drought stress followed by recovery. Plant Biotechnology Reports 1: 37–48.

[24] Pei ZM, Kuchitsu K, Ward JM, Schwarz M, Schroeder JI Differential abscisic acid regulation of guard cell slow anion channels in Arabidopsis wild-type and abi1 and abi2 mutants. Plant cell 9(3): 409–423. 9090884 10.1105/tpc.9.3.409 PMC156927

[25] Bates L, Waldren RP, Teare ID (1973) Rapid determination of free proline for water stress studies. Plant and Soil 39: 205–207.

[26] Arnon DI (1949) Copper enzymes in isolated chloroplasts, polyphenoxidase in beta vulgaris. Plant physiology 24: 1–15. 16654194 10.1104/pp.24.1.1 PMC437905

[27] Xiong L, Ishitani M, Lee H, Zhu JK (2001b) The *Arabidopsis LOS5/ABA3* locus encodes a molybdenum cofactor sulfurase and modulates cold and osmotic stress responsive gene expression. Plant cell 13: 2063–2083. 11549764 10.1105/TPC.010101 PMC139452

[28] Livak KJ, Schmittgen TD (2001) Analysis of Relative Gene Expression Data Using Real-Time Quantitative PCR and the 2DDCT Method. METHODS 25: 402–408. 11846609 10.1006/meth.2001.1262

[29] Gao X, Britt RC, Shan L, He P (2011) Agrobacterium-Mediated Virus-Induced Gene Silencing Assay In Cotton. J. Vis. Exp 54: e2938. 10.3791/2938 PMC3217629 21876527

[30] Kobayashi Y, Murata M, Minami H, Yamamoto S, Kagaya Y (2005) Abscisic acid-activated SNRK2 protein kinases function in the gene-regulation pathway of ABA signal transduction by phosphorylating ABA response element binding factors. Plant J 44: 939–949. 16359387 10.1111/j.1365-313X.2005.02583.x

[31] Zhang H, Mao X, Wang C, Jing R (2010) Overexpression of a common wheat gene *TaSnRK2.8* enhances tolerance to drought, salt and low temperature in *Arabidopsis* . PLoS ONE 5: e16041. 21209856 10.1371/journal.pone.0016041 PMC3012728

[32] Hawes MC, Gunawardena U, Miyasaka S, Zhao X (2000) The role of root border cells in plant defense. Trends Plant Sci 5: 128–133. 10707079 10.1016/s1360-1385(00)01556-9

[33] Dainty J (1976) Water relations of plant cells. *In* APGottingen, MHZimmermann, eds, Encyclopedia of Plant Physiology, Vol 2 . New Series, Part A. Springer-Verlag, Berlin, pp 12–35.

[34] Levitt J (1980) Response of plant to Environmental Stress, water, radiation, salt and other stresses. Academic press, New York.

[35] Steponkus PL (1984) Role of the plasma membrane in freezing injury and cold acclimation. Annu. Rev. Plant Physiol 35: 543–584.

[36] Steponkus PL, Uemura M, Webb MS (1993) A contrast of the cryostability of the plasma membrane of winter rye and spring oat-two species that widely differ in their freezing tolerance and plasma membrane lipid composition. In: Steponkus P L, editor. Adv. Low-Temperature Biol. Vol. 2 . London: JAI Press pp. 211–312.

[37] Claussen W (2005) Proline as a measure of stress in tomato plants. Plant Sci 168: 241–248.

[38] Younis ME, Hasaneen MNA, Tourky MNS (2009) Plant growth, metabolism and adaptation in relation to stress conditions. XXIV. Salinity biofertility interactive effects on proline, glycine and various antioxidants in Lactuca sativa. Plant Omics J 2: 197–205.

[39] Zhu JK (2002) Salt and drought stress signal transduction in plants. Annu Rev Plant Biol 53: 247–273. 12221975 10.1146/annurev.arplant.53.091401.143329 PMC3128348

[40] Granier C, Tardieu F (1999) Water deficit and spatial pattern of leaf development variability in responses can be simulated using a simple model of leaf development. Plant Physiol 119: 609–620. 9952457 10.1104/pp.119.2.609 PMC32138

[41] Wang ZQ, Yuan YZ, Ou JQ, Lin QH, Zhang CF (2007) Glutamine synthetase and glutamate dehydrogenase contribute differentially to proline accumulation in leaves of wheat (*Triticum aestivum*) seedlings exposed to different salinity. Plant Physiol 164: 695–701. 10.1016/j.jplph.2006.05.001 16777263

[42] Pareek A, Sopory SK, Bohnert HJ, Govindjee EDS (2010) Abiotic Stress Adaptation in Plants: Physiological, Molecular and Genomic Foundation, Springer, Dordrecht, pp. 283e305.

[43] Everard JD, Gucci R, Kahn JA, Flore WH (1994) Gas exchange and carbon partitioning in the leaves of celery (Apium graveolens L.) at various levels of root zone salinity, Plant Physiol. 106: 281e292. 10.1104/pp.106.1.281 PMC159526 12232328

[44] Schroeder JI, Kwak JM, Allen GJ (2001) Guard cell abscisic acid signalling and engineering drought hardiness in plants. Nature 410: 327–330. 11268200 10.1038/35066500

[45] Yoshida R, Hobo T, Ichimura K, Mizoguchi T, Takahashi F (2002) ABA-activated SnRK2 protein kinase is required for dehydration stress signaling in Arabidopsis Plant Cell Physiol. 43: 1473–1483. 10.1093/pcp/pcf188 12514244

[46] Mustilli AC, Merlot S, Vavasseur A, Fenzi F, Giraudat J (2002) Arabidopsis OST1 Protein Kinase Mediates the Regulation of Stomatal Aperture by Abscisic Acid and Acts Upstream of Reactive Oxygen Species Production. Plant Cell 14: 3089–3099. 12468729 10.1105/tpc.007906 PMC151204

[47] Chinnusamy V, Jagendorf A, Zhu JK (2005) Understanding and improving salt tolerance in plants, Crop Sci. 45: 437e448.

[48] Hu H, Dai M, Yao J, Xiao B, Li X, et al (2006) Overexpressing a NAM, ATAF, and CUC (NAC) transcription factor enhances drought resistance and salt tolerance in rice. Proc Natl Acad Sci USA 103: 12987–12992. 16924117 10.1073/pnas.0604882103 PMC1559740

[49] Ko JH, Yang SH, Han KH (2006) Upregulation of an Arabidopsis RING-H2 gene, *XERICO*, confers drought tolerance through increased abscisic acid biosynthesis. Plant J 47: 343–355. 16792696 10.1111/j.1365-313X.2006.02782.x

[50] Hiroaki F, Paul E, Jian-Kang Z (2007) Identification of Two Protein Kinases Required for Abscisic Acid Regulation of Seed Germination, Root Growth, and Gene Expression in *Arabidopsis* . The Plant Cell 19 (2): 485–494. 17307925 10.1105/tpc.106.048538 PMC1867333

[51] Riichiro Y, Tokunori H, Kazuya I, Tsuyoshi M, Fuminori T, et al (2002) ABA-Activated SnRK2 Protein Kinase is Required for Dehydration Stress Signaling in *Arabidopsis* . *Plant Cell Physiol* 43 (12): 1473–1483. 12514244 10.1093/pcp/pcf188

[52] Sang-Youl P, Pauline F, Noriyuki N, Davin RJ, Hiroaki F, et al (2009) Abscisic Acid Inhibits Type 2C Protein Phosphatases via the PYR/PYL Family of START Proteins. Science 324: 1068. 19407142 10.1126/science.1173041 PMC2827199

[53] Kavi PB, Zonglie H, Cuo-Hua M, Chein-An AH, DeshPal SV (1995) Overexpression of A1-Pyrroline-5-Carboxylate Synthetase lncreases Proline Production and Confers Osmotolerance in Transgenic Plants Plant Physiol. 108: 1387–1394. 10.1104/pp.108.4.1387 PMC157516 12228549

[54] Lopez-Molina L, Mongrand S, McLachlin DT, Chait BT, Chua NH (2002) ABI5 acts downstream of ABI3 to execute an ABA-dependent growth arrest during germination. Plant J 32: 317–328. 12410810 10.1046/j.1365-313x.2002.01430.x

[55] Ratcliff F, Martin-Hernandez AM, Baulcombe DC (2001) Tobacco rattle virus as a vector for analysis of gene functions by silencing. Plant J 25: 237–245. 11169199 10.1046/j.0960-7412.2000.00942.x

[56] Liu Y, Schiff M, Dinesh-Kumar S (2002) Virus-induced gene silencing in tomato. Plant J 31: 777–786. 12220268 10.1046/j.1365-313x.2002.01394.x

[57] Cui L, Jian-Min Y, Yun-Zhou L, Zhen-Cai Z, Qiao-Li W, et al (2013) Silencing the *SpMPK1*, *SpMPK2*, and *SpMPK3* Genes in Tomato Reduces Abscisic Acid—Mediated Drought Tolerance Int. J. Mol. Sci 14: 21983–21996. 10.3390/ijms141121983 PMC3856046 24201128

[58] Senay S, Fikret Y, Sebnem K, Sebnem E (2011) The effect of salt stress on growth, chlorophyll content, lipid peroxidation and antioxidative enzymes of pumpkin seedling. African Journal of Agricultural Research 6(21): 4920–4924.

[59] Yasar F, Ellialtioglu S, Yildiz K (2008) Effect of salt stress on antioxidant defense systems, lipid peroxidation, and chlorophyll content in green bean. Russian J. Plant Physiol 55(6): 782–786.

