# Supplementary figures and images for: Cloning of Gossypium hirsutum Sucrose Non-Fermenting 1-Related Protein Kinase 2 Gene (GhSnRK2) and Its Overexpression in Transgenic Arabidopsis Escalates Drought and Low Temperature Tolerance
Source: PLoS One. 2014 Nov 13;9(11):e112269. doi: 10.1371/journal.pone.0112269 (PMC4231032; doi:10.1371/journal.pone.0112269)

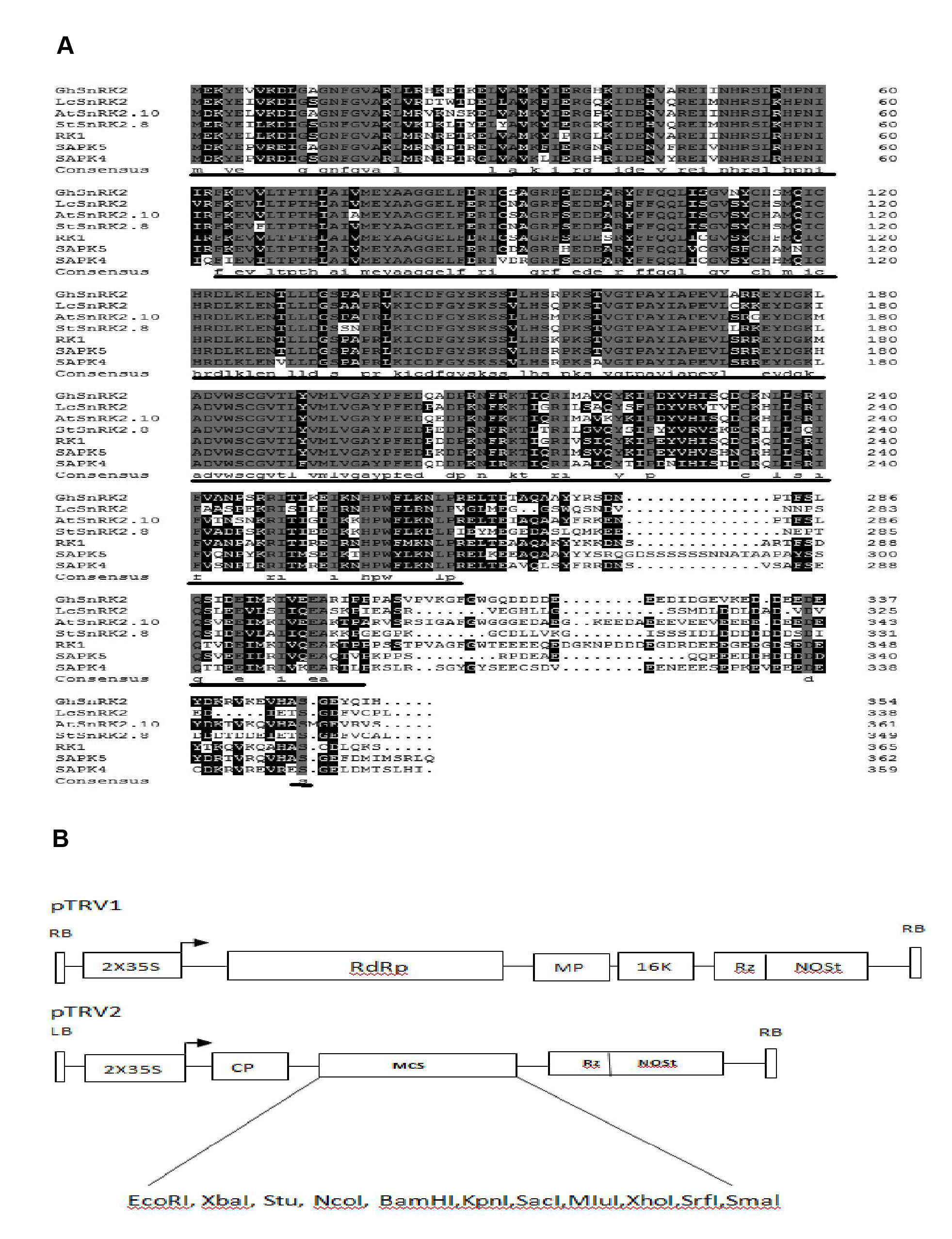

Supplement: Figure S1 — Multiple sequence alignment of GhSnRK2 and closely related SnRK2s from other plants species and VIGS construct. (A) Alignment of GhSnRK2 and closely related SnRK2s. The relatively conserved motif is underlined. The deduced amino acid sequence displays relatively high homology with the monocot SnRK2 family members Oryza sativa (RK1), ABB89146 and with the dicot species AtSnRK2.10, AEE33751.1. (B) Virus-induced gene silencing construct. The TRV VIGS vectors were modified based on a pTRV1 containing RNA-dependent RNA polymerase (RdRp), movement protein (MP), a 16 kDa cysteine-rich protein (16K), CaMV 35S promoters (2X35S) and a NOS terminator (NOSt) T-DNA vector. pTRV2 contains the coat protein (CP), multiple cloning sites (MCSs), CaMV 35S promoters (2X35S) and a NOS terminator (NOSt) T-DNA vector. Both vectors contain Rz, which is designated as a self-cleaving ribozyme, LB (left border) and RB (right border) of the T-DNA. (TIF) [file pone.0112269.s001.tif]

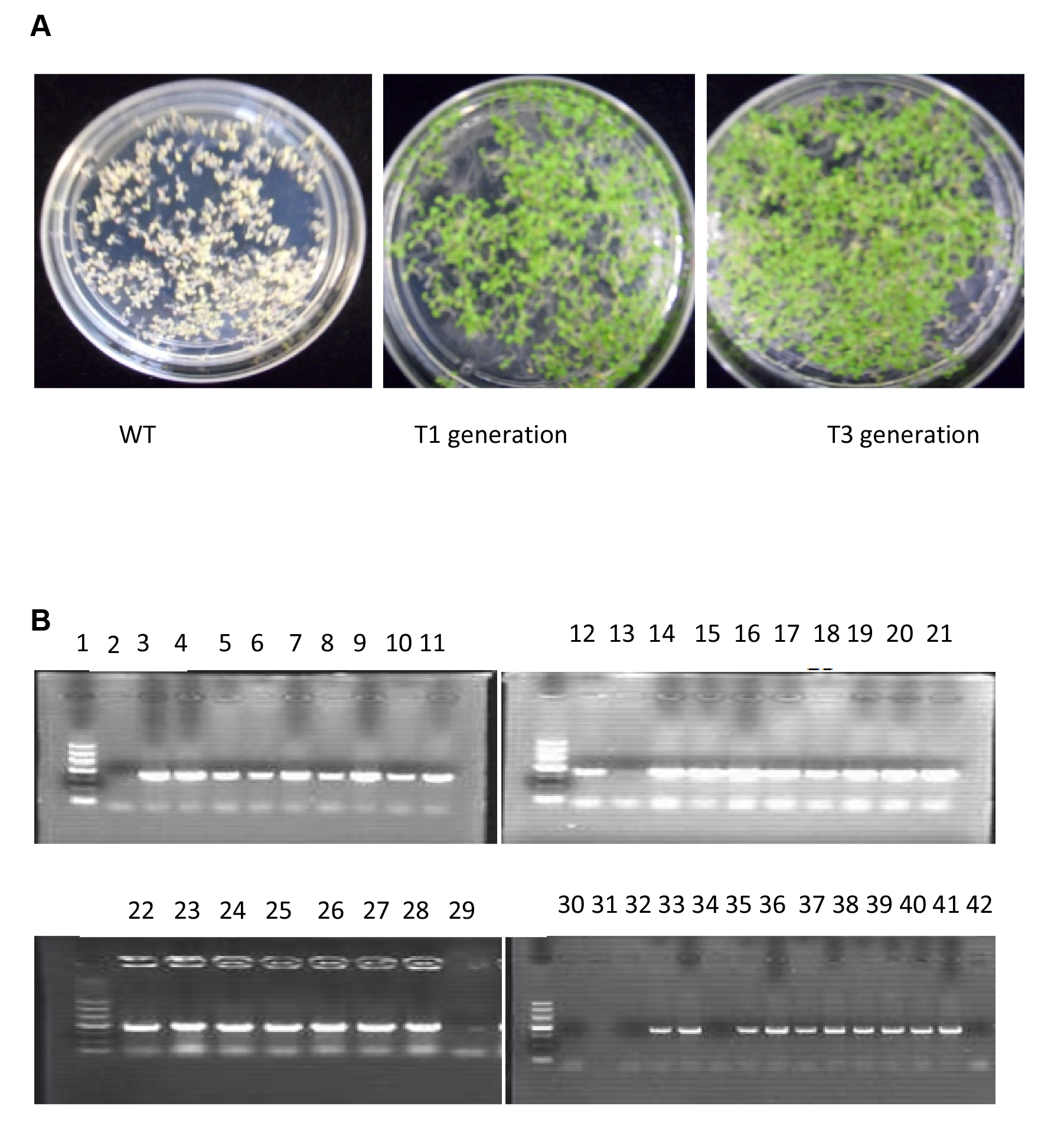

Supplement: Figure S2 — Survival rate in kanamycin-containing medium and PCR confirmation of GhSnRK2 gene expression in transgenic Arabidopsis. (A) The survival rate of WT and GhSnRK2 transgenic plants in MS medium supplemented with the antibiotic kanamycin. Photograph of a representative plant was captured after 9 days of germination in kanamycin-containing medium. (B) Confirmation of GhSnRK2 gene in transgenic Arabidopsis. Genomic DNA from the first generation of the plants (T1) was extracted and used as a template for gene-specific primers. Lane 1: DNA molecular marker III standard; lane 2: negative control. (TIF) [file pone.0112269.s002.tif]
