# Supplementary material for: Cloning of Gossypium hirsutum Sucrose Non-Fermenting 1-Related Protein Kinase 2 Gene (GhSnRK2) and Its Overexpression in Transgenic Arabidopsis Escalates Drought and Low Temperature Tolerance
Source: PLoS One. 2014 Nov 13;9(11):e112269. doi: 10.1371/journal.pone.0112269 (PMC4231032; doi:10.1371/journal.pone.0112269)
Supplement: Table S1 — Survival (%) of GhSnRK2 transgenic plants in kanamycin-containing medium. At T1 generation, the ratio of dead to surviving plant was approximately 1∶3 in kanamycin-containing LB medium. The survival rate at T3 generation was 100% in kanamycin-containing LB medium. The values are expressed as the mean germination rate (%) of approximately 200 seeds. (DOCX) [file pone.0112269.s003.docx]

**Table S1. Survival (%) of *GhSnRK2* transgenic plants in kanamycin-containing medium.**

| Representative Lines | T1 generation  Survival (%) | T1 generation  Dead (%) | T3 generation  Survival (%) |
| --- | --- | --- | --- |
| 1 | 70 | 30 | 100 |
| 2 | 72 | 28 | 100 |
| 3 | 68 | 32 | 100 |
| 4 | 70 | 30 | 100 |
| 5 | 66 | 34 | 100 |
