# Supplementary material for: Cloning of Gossypium hirsutum Sucrose Non-Fermenting 1-Related Protein Kinase 2 Gene (GhSnRK2) and Its Overexpression in Transgenic Arabidopsis Escalates Drought and Low Temperature Tolerance
Source: PLoS One. 2014 Nov 13;9(11):e112269. doi: 10.1371/journal.pone.0112269 (PMC4231032; doi:10.1371/journal.pone.0112269)
Supplement: Table S2 — The lists of primers sequences used in this study. (DOCX) [file pone.0112269.s004.docx]

**Table S2. Primers used in this study**

| Primer name | Primer Sequence 5’-3’ | Function |
| --- | --- | --- |
| *GhSnRK2* | F: GTGGTTTTGACGCCTACCCATTTA  R: AGAAAATGTAGGGTTATCGCTCCG | qRT-PCR |
| *GhSnRK2* | F:TCTAGAATGGAGAAATATGAGGTGGT  R: GGATCCTTAATGGATTTGATATTCTC | Amplification of ORF |
| *AtP5CS1* | F: GAGCAATGGAGTCACTTTGTATGG  R: TTCCTCTCATTATCCATCTCGTTG | qRT-PCR |
| *AtABI5* | F: AATAAGAGAGGGATAGCGAACGAG  R: CTTGGTGAAGGCTGGTGTGGTTAG | qRT-PCR |
| *AtABI3* | F: GATAGCAACGAGTTCTTTGACACC  R: CACCACCAACCTCTCTTATCAACA | qRT-PCR |
| *AtRD29A* | F: AACGAGGGGAAGATAAAAGTGTGTC  R: AGGCTTTGTCTTCTTCTTCAGTTGTC | qRT-PCR |
| *AtRD29B* | F: CGACAAGAGGTGATGTGAAAGTAGA  R: CTCAGAATCATCTCTCTCTTTGGGAC | qRT-PCR |
| *AtCBF1* | F: GGATGCCGACTTTGTTGGATAATA  R: TAGTAACTCCAAAGCGACACGTCA | qRT-PCR |
| VIGS | F: TCTAGACCTTGGTTTCTGAAGAACTT  R: GATCCATGGATTTGATATTCTCCAC | Amplification of specific fragment |
| VIGS | F: GAACTTGCCCAGAGAGCTAACAGA  R: GTCGTCTTCTTTCACTTCCCCATC | qRT-PCR |
